# Recessive variants in ESCRT-II component VPS25 alter vesicular transport and small extracellular vesicle processing in lethal neonatal encephalopathy

**DOI:** 10.1186/s13073-026-01693-z

**Published:** 2026-09-14

**Authors:** Ioannis Isaioglou, Lama AlAbdi, Muhammad Tehseen, Yossef Lopez de los Santos, Mansour Aldehaiman, Gloria Lopez-Madrigal, Norah Altuwaijri, Viviana Hermosilla Aguayo, Anna Pleyers, Maya Ayach, Ashraf Al-Amoudi, Jing Kai, Vlad-Stefan Raducanu, Bader Alhaddad, Edda Hofstätter, Amani Al-Amodi, Mais O. Hashem, Hessa S. Alsaif, Claudius Fazelnia, Firdous Abdulwahab, Amal Jaafar, Tarfa Alshidi, Rana Helaby, Adriana M. Montaño, Kara C. Klemp, Ellen Totten, Wesam Kurdi, Saly Eltohamy, Hadeel Abdollah E. Alrukban, Bashayer Alawam, Licia Selleri, Davor Lessel, Dimitrios Kapogiannis, Samir M. Hamdan, Stephen R. Braddock, Fowzan S. Alkuraya, Jasmeen S. Merzaban

**Affiliations:** 1 Biomedical Sciences Division, King Abdullah University of Science and Technology (KAUST), Thuwal, 23955-6900 Saudi Arabia; 2 Department of Translational Genomics, Genomic Medicine Centre of Excellence, King Faisal Specialist Hospital and Research Center, Riyadh, Saudi Arabia; 3 Program in Craniofacial Biology, Department of Orofacial Sciences, Department of Anatomy, Institute for Human Genetics, Eli and Edythe Broad Center of Regeneration Medicine and Stem Cell Research, University of California, San Francisco, San Francisco, CA USA; 4 Institute of Human Genetics, University Hospital Salzburg, Paracelsus Medical University, Salzburg, 5020 Austria; 5 KAUST Core Labs, KAUST, Thuwal, 23955-6900 Kingdom of Saudi Arabia; 6 Lifera Omics, Lifera Omics Innovation Boulevard St, KAFD, Al Aqeeq Dist., Riyadh, 13519 Saudi Arabia; 7 Division of Neonatology, Department of Pediatrics and Adolescent Medicine, Paracelsus Medical University, Salzburg, 5020 Austria; 8 Wellness and Preventative Medicine Institute, Health Sector, King Abdulaziz City for Science and Technology (KACST), Riyadh, 11442 Saudi Arabia; 9 Department of Obstetrics and Gynecology, Paracelsus Medical University, Salzburg, 5020 Austria; 10 Department of Pediatrics, and Biochemistry and Molecular Biology, School of Medicine, Saint Louis University, St. Louis, MO USA; 11 Department of Pediatrics, Saint Louis University School of Medicine, St Louis, MO USA; 12 Department of Obstetrics and Gynecology, King Faisal Specialist Hospital and Research Center, Riyadh, Saudi Arabia; 13 Dr. Sulaiman Al Habib Medical Group, Riyadh, Saudi Arabia; 14 Department of Pediatrics, Prince Sultan Military Medical City, Riyadh, Saudi Arabia; 15 Department of Pediatrics, King Fahad Hospital of the University, Al-Khobar, Saudi Arabia; 16 Institute of Human Genetics, University of Regensburg, Regensburg, 93053 Germany; 17 Institute of Clinical Human Genetics, University Hospital Regensburg, Regensburg, 93053 Germany; 18 Laboratory of Clinical Investigation, Intramural Research Program, National Institute on Aging, National Institutes of Health (NIA/NIH), Baltimore, MD USA; 19 Department of Neurology, Johns Hopkins School of Medicine, Baltimore, MD USA; 20 College of Medicine, Alfaisal University, Riyadh, Saudi Arabia; 21 KAUST Center of Excellence for Smart Health (KCSH), King Abdullah University of Science and Technology (KAUST), Thuwal, Saudi Arabia

**Keywords:** ESCRT-II, VPS25, Small extracellular vesicles, Exosomes, Protein cargo, Lysosomes, MVBs, Brain anomalies, Hydrocephalus, Corpus callosum

## Abstract

**Background:**

The role of small extracellular vesicles (sEVs), such as exosomes, in cell-to-cell communication is increasingly recognized. Both during development and postnatally, sEVs have been shown to mediate a multitude of essential functions in the central nervous system. While abnormal sEV biogenesis has been observed in a number of neurodevelopmental disorders, a causal link has been largely lacking.

**Methods:**

We clinically evaluated five families in which newborns presented with a novel neonatal-onset encephalopathy. Positional mapping, exome sequencing, and trio exome sequencing were utilized to identify the underlying genetic variants. Additionally, we utilized *Vps25* hypomorphic mouse mutants and performed cellular assays to evaluate ESCRT-II interaction, cellular localization, and sEV secretion and cargo capacities.

**Results:**

The observed lethal encephalopathy is characterized by severe hypotonia, brain anomalies, and epilepsy. In families 1, 4, and 5 (multiplex consanguineous), we identified a novel homozygous missense variant in *VPS25* as the likely candidate. Family 2 comprises a simplex case with two missense variants in trans in *VPS25*, one of which impacts the same residue as in the other three families. Family 3 also comprises a simplex case where we revealed a homozygous missense variant. VPS25 is an essential component of ESCRT-II, itself required for sEV biogenesis. We show that *Vps25* hypomorphic mouse mutants recapitulate the developmental brain malformation observed in patients. We also show that mutant VPS25 is associated with abnormal ESCRT-II interaction, abnormal cellular localization, and highly abnormal sEVs. Specifically, mutant cells displayed engorged multivesicular bodies and were significantly defective in their capacity to secrete sEVs such as exosomes, while secreted sEVs contained altered cargo, lacking vital proteins for axon protection and maintenance.

**Conclusions:**

We report a novel neurodevelopmental disorder caused by biallelic hypomorphic variants in *VPS25*. The consistent impact of these variants on sEV biogenesis suggests a potential pathomechanism. While future cases will further delineate the phenotypic spectrum, these findings offer a new perspective on abnormal sEVs as drivers of human neurodevelopmental disorders.

**Supplementary Information:**

The online version contains supplementary material available at https://doi.org/10.1186/s13073-026-01693-z.

## Background

Many fundamental processes in the cell related to catalyzing the budding and scission of membranes, such as the formation of multivesicular bodies (MVBs) and cytokinesis, rely on the evolutionarily conserved endosomal sorting complexes for transport (ESCRT) [1]. The ESCRT-II complex is well-known for its role in mediating the sorting of ubiquitinated membrane proteins into the MVB pathway, which ultimately leads to the delivery of these proteins to lysosomes and/or extracellular vesicles [1–3].

One critical component of the ESCRT-II complex is the 176-amino-acid protein known as vacuolar protein-sorting-associated protein 25 (VPS25). The ESCRT-II complex is a hetero-tetrameric protein complex consisting of two copies of the VPS25 and a single copy each of the VPS22 and VPS36 proteins [2]. Recently, cases of severe developmental and epileptic encephalopathy, were reported as a result of bi-allelic mutations in VPS22 [4]. Additionally, mutants of *VPS25* orthologs have been linked to various phenotypic anomalies in different species, such as *Saccharomyces cerevisiae* [5], *Drosophila melanogaster* [6] and *Mus musculus* [7]. In mice, Vps25^null^ embryos were lethal by E8.5 and even a hypomorphic line could not survive beyond E16.5 and displayed abnormalities in lysosomal and MVB functions [7]. More specifically, lysosome trafficking was impaired, leading to a reduction in cargo degradation, while MVBs showed significant increases in their diameters as engorged late endosomes [7].

Small Extracellular Vesicles (sEVs), such as exosomes, are membranous vesicles with a typical diameter of 40–200 nm containing protein, RNA, and lipid molecules as their cargo [8]. Exosome biogenesis occurs inside endosomes and is mediated through the inward budding of the membrane of an early endosome, which subsequently turns into an MVB [9]. MVB biogenesis occurs through at least two mechanisms, one of which is dependent on ESCRT complexes [9–11]. These MVBs subsequently fuse with lysosomes, promoting their destruction or, alternatively, can fuse with the cell membrane and release their content into the extracellular space as exosomes. Indeed, studies have illustrated that targeting the ESCRT pathway represses the biogenesis and secretion of exosomes, emphasizing the critical role that the ESCRT pathway plays in the generation of such extracellular vesicles [11–13].

The role of sEVs in brain development remains poorly understood. Recent evidence suggests that sEVs are critical for neural progenitor proliferation, neuronal differentiation, and neuron-astroglia communication [14, 15]. The relevance of these recent findings to neurodevelopmental disorders is unclear since research into the neuronal function of sEVs has been largely directed at adult neurodegenerative diseases [16]. In this study, we describe a novel neurodevelopmental disorder characterized by severe encephalopathy with neonatal onset, early lethality, and associated brain anomalies that are recapitulated by the mouse model. We show that this syndrome is associated with biallelic variants in *VPS25*, a major component of ESCRT-II, and that patient cells display abnormal sEV turnover.

## Methods

### Human subjects

This study includes a cohort of five unrelated families presenting with a novel neonatal-onset encephalopathy characterized by severe hypotonia, brain anomalies, and early mortality. The total study cohort comprised a total number of seven affected individuals, not counting deceased individuals who were not available for testing. Families 1, 4, and 5 are multiplex consanguineous families of Middle Eastern descent. Family 1 was recruited for research exome sequencing as part of a large Mendelian Genomics Program in Saudi Arabia. Families 4 and 5 were identified by querying the Lifera Omics Database (LODB), a repository of > 50,000 clinical exomes and genomes from the Middle East [17]. Families 2 and 3 comprise simplex cases from North America and Europe, respectively. Both families underwent clinical trio exome sequencing by independent CLIA-accredited laboratories. Following non-diagnostic results, the respective clinical labs uploaded the variants to GeneMatcher, which facilitated their inclusion in this study cohort. Detailed clinical data for all families were collected by board-certified clinical geneticists. For functional downstream analyses, venous blood samples and skin punch biopsies were collected to establish lymphoblastoid cell lines (LCLs) and primary fibroblast cultures, respectively. Specifically, fibroblasts were established from Family 2 patient, and LCLs were established from Families 1, 2, and 4. For Family 3, clinical and genomic data were included in the study; however, biological samples for functional analysis were not obtained as the legal guardians did not provide consent for the use of patient-derived cell lines. Wild-type LCL utilized as controls were collected from a 2-year-old male (WT1), a 3-year-old female (WT2), and a 6-year-old female (WT3). Wild-type control fibroblasts were established from tissue obtained during neonatal circumcisions. Informed consent was obtained from Families 1, 2, 4, and 5 as part of an IRB-approved research protocol (KFSHRC RAC# 2121053, 2210029, and 2080006). For Family 3, the institutional review board approval was EK 1115/2024.

### Exome sequencing, autozygosity mapping and variant interpretation

Combining exome sequencing and autozygosity mapping in Families 1, 4, and 5 was as described before [18]. Briefly, genome-wide genotyping was conducted on the Axiom platform in accordance with the manufacturer’s guidelines. Regions of homozygosity (ROH) ≥ 2 Mb were identified and used as proxies for autozygosity using the autoSNPa tool (https://dna-leeds.co.uk/autosnpa/). Exome sequencing and subsequent filtering of candidate variants based on autozygome coordinates were performed as described previously [18]. Exome sequencing in Families 2 and 3 was performed by two independent CLIA-accredited laboratories. Variants were interpreted according to the ACMG guidelines [19].

### Histology of *Vps25* hypomorphic mutant mouse embryos

*Vps25*^*+/+*^ and *Vps25*^*ENU/ENU*^ embryos [7, 20] were retrieved from pregnant dams at gestational day (E) 14.5. The Vps25^*ENU*^ mutation corresponds to an N-ethyl-N-nitrosourea (ENU)-induced G-to-A transition within the third intron of *Vps25*, resulting in an in-frame 27-nucleotide insertion in the mRNA that is translated into nine additional neutral amino acids. Following retrieval, embryos were fixed overnight in 4% paraformaldehyde at 4° C, dehydrated in serial alcohols (1 × 70%, 1 × 80%, 1 × 90%, 2 × 96%, 2 × 100% ethanol, followed by 1 × 100% isopropanol for 20 min each), cleared twice for 30 min with Histo-Clear II (HS-202, National Diagnostics) and embedded in paraffin. 10 μm-thick head coronal sections were obtained with a Leica Biosystems RM2245 Semi-Automated Rotary Microtome and mounted on glass slides. Sections were de-waxed, rehydrated, and stained with Trichrome stain kit (ab150686, Abcam) following the manufacturer’s instructions. Briefly, slides were submerged in preheated Bouin’s Fluid (56–64 °C) for 60 min. After 10 min of cooling at room temperature, sections were rinsed in tap water, followed by distilled water. Next, sections were stained for 5 min in Weigert’s Iron Hematoxylin and rinsed with running tap water. Slides were sequentially submerged for 15 min in Biebrich Scarlet / Acid Fuchsin solution, 15 min in Phosphomolybdic/Phosphotungstic Acid solution and 10 min with Aniline Blue solution, rinsing with distilled water after every staining step except after the Phosphomolybdic/ Phosphotungstic Acid solution. Slides were subjected to a final rinse for 5 min with 1% acetic acid solution. Stained sections were dehydrated in serial alcohols, cleared twice in Histo-Clear II for 5 min, and mounted with Omnimount (HS-110, National Diagnostics). Images were obtained using a Leica M205 FA stereo microscope.

### Structural biology evaluation of the mutant ESCRT-II complex

The ESCRT-II template was retrieved from PDB (3CUQ [21]). The complex structure had low resolution and weak PDB scores. QMEAN assessment showed deviations from best protein models in PDB [22, 23]. Model refinement was crucial for mutation analysis in VPS25. Refinement used the same pdb for homology modelling. MDs with YASARA2 force field refined the model. SolvX, Ramachandran analysis, PROCHECK, and Molprobity were used for assessment. The best ESCRT-II complex was chosen for further analysis [24–27].

YASARA2 force field and Moldock scoring function were used to study the effect of VPS25 mutation on ESCRT-II complex stability [24, 28]. Models of P8S, P8L, and G172S mutations were energetically minimized using YASARA2 force field [24, 29].

### Steered Molecular Dynamics simulations (SMDs)

Steered Molecular Dynamic simulations was performed in order to evaluate the effect of the mutations in VPS25 on the resilience of the ESCRT-II complex over time. The ESCRT-II SMDs were run with YASARA [30]. The setup included the hydrogen bonding network optimization [31] as well as the pKa prediction to optimize the protonation states at 7.4pH [32]. NaCl ions were added with physiological concentration of 0.9%. After steepest descent and simulated annealing minimizations to remove clashes, the force field used was AMBER14 [33] For the solute, GAFF2 and AM1BCC for ligands, and TIP3P for water [34, 35]. Cutoff was 8 Å for Van der Waals forces, no cutoff for electrostatic forces [36]) and no cutoff was applied to electrostatic forces (using the Particle Mesh Ewald algorithm [37]). Equations of motions integrated with multiple timesteps at 298 K and 1 atm pressure. After 3 ps equilibration, minimum acceleration of 2000 pm/ps^2 applied to ligand atoms. Pulling force estimated using ligand mass. Pulling direction updated continuously. Simulation stopped when ligand moved 15 Å away. Peak pulling force and total work calculated. Simulations performed three times per case. These simulations were performed three times per case by changing the seeding parameters of each simulation.

### Molecular Dynamic (MD) simulations

Longer timescale MDs were performed with the same complexes and parameters as SMDs, excluding pulling forces. Simulation systems reached equilibrium fast (5-7ns). First 25% of simulation time (~ 50ns) considered equilibrium phase. Due to large number of atoms, runs performed in duplicates. Time scale explored was 200ns, with randomized initial seeds. Single runs MDs (200ns) performed on non-deleterious mutations in VPS25 (K45Q, V167I, I76V) from gnomAD database [38]. YASARA Structure suite used for running and analysis [30]. UCSF Chimera 1.16 and InkScape 1.0.1 used for preparing structural images [39]. Consurf-DB used to estimate evolutionary conservation score of amino acids in ESCRT-II protein. Secondary structure images obtained from PDBSum webserver for 3CUQ pdb file [40, 41].

### Cell culture

Transformed lymphoblasts and primary fibroblasts derived from the patients were cultured into a humidified incubator under standard conditions (37 °C & 5% CO_2_) in a concentration of 0.7-1 × 10^6^ cells/mL. Culture media for the lymphoblasts was Roswell Park Memorial Institute media (RPMI 1640, Thermo Fisher) containing 15% Fetal Bovine Serum (FBS, Gibco), 1% HyClone Penicillin-Streptomycin (Sigma-Aldrich) and 1% L-glutamine (Gibco), while culture media for the fibroblasts was Minimum Essential Media (MEM-α, Thermo Fisher) containing 15% FBS, 1% HyClone Penicillin-Streptomycin and 1% L-glutamine.

### Cell lysis

The lysis buffer contained: 1% (v/v) Triton™ X-100 (Electrophoresis, Fisher BioReagents™), 150mM NaCl, 50mM Tris-HCl (pH.7.6), 1µM Phenylmethane Sulfonyl Fluoride (PMSF), 1% (v/v) Halt™ Phosphatase Inhibitor (Thermo Scientific) and 1% Pierce Protease Inhibitor tablets, EDTA free (Thermo Fisher). For each million of cells, 20µL of lysis buffer were used. The mixture was incubated at 4 °C, rotating for one hour and thereafter was centrifuged for 30 min at 16,100 g, at 4 °C. The resulting supernatant was the total lysate and was used for downstream analysis. When needed, cell lysates were balanced based on BCA method (Thermo Fisher).

In assays where the protein-protein interactions needed to be maintained, such as size exclusion chromatography and Co-Immunoprecipitation, Triton™ X-100, NaCl and Tris-HCl were replaced by CHAPS buffer (FIVEphoton Biochemicals™).

### Western blot

Samples were run on Criterion TGX Precast Protein Gels of 4%–20% polyacrylamide gels (BioRad), followed by transfer, using the “sandwich” set up, to a 0.45 μm PVDF membrane (Merck). After eight hours blocking with 5% (w/v) Bovine Serum Albumin (Sigma-Aldrich), the membranes were incubated overnight with the respective primary antibody (Additional file 1: Table S1), followed by three washing steps and thereafter a 45-minute incubation with the respective secondary antibody. After three washes, the membranes were imaged using the ECL Prime western Blotting System (Merck) reagent, into the ChemiDoc™ MP Imaging System (Bio-Rad).

### ESCRT II expression and purification

MultiBac™ expression system (Geneva Biotech) was used to express the human ESCRT II complex in Sf9 insect cells. For this purpose, VPS22 (accession no. Q96H20) and VPS25 or VPS25 mutant (P8S or M49V)(accession no. Q9BRG1) containing Twin-Strep-Tag and TEV protease cleavage sequence at the N-terminus, and VPS36 (accession no. Q86VN1) containing eight histidine residues at the C-terminus were cloned into pACEBac1 (Gent^+^) along with independent promoters and terminators by the GenScript. Bacmid DNA was generated by transforming the recombinant vector containing all three subunit gene expression cassettes into DH10MultiBac™.

To prepare the baculovirus, the bacmid DNA of ESCRT II wild type (*VPS25*^WT^) and ESCRT II mutant (*VPS25*^P8S^) were transfected separately into Sf9 cells using FuGENE^®^ HD (Promega) according to the manufacturer’s instructions. Resulting supernatant was obtained as the P1 virus stock which was then amplified to obtain the P2 virus stock. P2 virus stock was further amplified to obtain P3 virus stock for large-scale expression. ESCRT II wild type (VPS25^WT^) and ESCRT II mutant (VPS25^P8S^ or VPS25^M49V^) were expressed separately by transfecting 2.8 L of suspension culture of Sf9 cells at 2 × 10^6^ cells/ml with P3 virus stock for 72 h. Cells were harvested by centrifugation at 5,500 xg for 10 min and re-suspended in 3 ml per 1 g of wet cells in 200 ml of lysis buffer [50 mM HEPES (pH 8), 500 mM NaCl, 20 mM Imidazole, 5mM β-Mercaptoethanol, 0.1% NP-40, 10% (v/v) Glycerol and EDTA free protease inhibitor cocktail tablet/50 ml (Roche, UK)]. Cells were sonicated and debris was removed by centrifugation at 95,834 xg for 1 h at 4 °C. The resultant supernatant was collected, filtered and directly loaded onto a HisTrap HP 1 ml affinity column (Cytiva) equilibrated with buffer A [50 mM HEPES (pH 8), 500 mM NaCl, 20 mM Imidazole, 5mM β-Mercaptoethanol and 10% (v/v) Glycerol]. After loading, the column was washed with 15 ml of buffer A followed by 10 ml elution gradient with buffer B [50 mM HEPES (pH 8), 400 mM NaCl, 500 mM Imidazole, 5mM β-Mercaptoethanol and 10% (v/v) Glycerol]. Peak fractions containing ESCRT II wild type (VPS25^WT^) and ESCRT II mutant (VPS25^P8S^ or VPS25^M49V^) were pooled separately and loaded directly onto StrepTrap XT 1 mL affinity (Cytiva) column equilibrated with buffer C [50 mM HEPES (pH 8), 300 mM NaCl, 5 mM β-Mercaptoethanol and 5% (v/v) Glycerol]. After loading, the column was washed with 15 ml of buffer C and the bound fractions were incubated for 2 h with 4 ml of 100% buffer D [50 mM HEPES (pH 8), 300 mM NaCl, 50 mM Biotin, 5 mM β-Mercaptoethanol and 5% (v/v) Glycerol] and eluted with 10 ml of 100% buffer D. Fractions that contained ESCRT II or ESCRT II P8S mutant were collected, concentrated, and loaded onto HiLoad™ 16/600 Superdex™ 200 pg (Cytiva) equilibrated with storage buffer [50 mM HEPES (pH 8), 200 mM NaCl, 1 mM DTT and 10% (v/v) Glycerol]. Fractions containing ESCRT II wild type (VPS25^WT^) and ESCRT II mutant (VPS25^P8S^ or VPS25^M49V^) were concentrated, flash-frozen and stored at -80 °C.

### Size exclusion chromatography

2 mL of total lysate from patients’ cells were loaded onto HiLoad™ 16/600 Superdex™ 200 pg (Cytiva) equilibrated with a buffer containing: 50 mM HEPES (pH 8), 200 mM NaCl, 1 mM DTT and 10% (v/v) Glycerol. From the resulted fractions, 100 µL were collected from the ones corresponding to fractions where the ESCRT-II complex was eluted in the purification experiment and thereafter, were prepared for western blot analysis.

### Co-immunoprecipitation

Six µg of VPS25 antibody (D-6 clone, Santa Cruz) attached to Protein G Dynabeads (Thermo Fisher) were incubated overnight rotating, with one mg of the respective, pre-cleaned, total lysate from patient-derived cells, at 4 °C. Thereafter, three washing steps using lysis buffer took place, followed by elution of the Co-Immunoprecipitation (CO-IP) product using a buffer containing 50% (v/v) NuPAGE^®^ LDS Sample Buffer (Thermo Fisher) and 50% PBS (v/v) in the presence of 100mM of DTT.

### Mass spectrometry

Sample preparation and data analysis was performed as described in our previous publication [42]. Briefly, in-gel digestion methodology was performed, using the Sequencing Grade Modified Trypsin (Promega). The resulting peptides were desalted using C18 pipette tips (Agilent) and were run into the timsTOF Pro (Bruker). The raw data were aligned to the UniProtKB/Swiss-Prot Human database using the Mascot (Matrix Science) and Compound Discoverer Software (Thermo Fisher). In silico analysis was performed using the STRING [43], DAVID [44, 45] and SRplot [46] online tools.

For sEV analysis, 1 × 10^10^ sEVs from each condition were lysed and separated by polyacrylamide gel electrophoresis before in-gel digestion.

### Confocal microscopy

500,000 cells were attached to an 8 well glass slide that was incubated overnight with 2% Poly-L-ornithine in PBS at 4 °C. Cells were then fixed and permeabilized using the fix and perm kit (Thermo Fisher). Following 3 washes with PBS, cells were incubated with VPS25 (B-4 clone, Santa Cruz) and VPS36 (Polyclonal, Novus Biologicals) primary antibodies, followed by 3 washes with PBS and then incubated with AF647 and AF488 secondary antibodies (Thermo Fisher) for 1 h each at room temperature, respectively. Otherwise, cells were incubated with Alexa Fluor™ 647 Phalloidin (Thermo Fisher) for 1 h. After 3 washes with PBS, cells were incubated using DAPI for 10 min and then were washed with PBS. Cells were then covered with ProLong™ Gold (Thermo Fisher) antifade mountant and the slide was sealed then imaged using Leica SP8 confocal microscopy.

Actin filament parallelness was quantified in ImageJ using the “lpx_ij_plugins_s” plugin. Individual cell images were cropped then skeletonized using the above-mentioned plugin. Skeletonized images were then processed to provide “NormAvgRad”. “NormAvgRad” represents how parallel the actin fibers in each cell.

### Flow cytometry of lysosomes

Flow cytometry measurement was used to determine the amount of present lysosomes in the cell. 300,000 lymphoblasts from each sample were collected and incubated for 1 h with 100 nM of LysoTracker™ Green DND-26 (Thermo Fisher) at 37^o^C with 5% CO_2_. Cells were then washed and measured using flow cytometry.

### TEM

Samples were fixed in 2.5% Glutaraldehyde, 2% paraformaldehyde in 0.1 M Sodium Cadodylate buffer pH 7.4 (Electron microscopy sciences, Hatfield, PA) over night at 4 °C. They were postfixed in 1% Osmium tetroxide (Electron microscopy sciences, Hatfield, PA), 1.5% potassium Ferrocyanide for 1 h at room temperature followed by 2% osmium tetroxide in water for 30 min at room temperature. Following fixation with osmium, samples were after incubated with 1% Uranyl acetate for 12 h at 4 °C and dehydrated in graded alcohol using Acetone 70%, 90%, 96% and 100% (twice), each for 15 min. Finally, the samples were embedded in Embed812 (Electron microscopy sciences, Hatfield, PA) for 48 h at 60 °C. Ultrathin sections of 90 nm were made using the Ultamicrotome UC7 (Leica Microsystems). Sections were submitted to a final stain using Lead Citrate 3% (Electron microscopy sciences, Hatfield, PA) and imaged using Titan 80–300 (Thermo Fisher Scientific) operated at an acceleration voltage of 300 kV.

### sEVs isolation and quantification

Lymphoblasts derived from different patients were placed into fresh media containing 15% (v/v) of exosome-depleted FBS (Thermo Fisher). After 18 h of culturing, sEVs from the culture media were isolated using serial centrifugation steps [42] and their concentration and size distribution were quantified by Nanoparticle Tracking Analysis via the NanoSight LM20 (Malvern Panalytical). The Camera Shutter and Camera Gain were in Auto mode; 4 videos of 30 s each were captured. The total number of sEVs was divided by the total number of cells, divided by the total time-in hours- which the cells were in culture. The number of cells and their viability (> 95%) were calculated using an automated cell counter (Countess 3, Invitrogen). The resulting number was the estimated number of sEVs produced per cell per hour.

### Cryo-EM

100,000 sEVs from each sample were applied to glow-discharged (Quorum Glow Discharge Unit, 35 mA, 1 min) holey carbon grid (Quantifoil R2/2 on gold or copper 300 mesh support), blotted for 3.5 s with Vitrobot filter paper (Electron Microscopy Sciences), and plunge frozen into liquid ethane cooled by liquid nitrogen using a Vitrobot (Thermo Fisher Scientific) operated at 4 °C and 100% humidity. Samples were imaged on a Titan Krios G4 (Thermo Fisher Scientific) operated at 300 kV and equipped with Falcon 4i direct electron detector (Thermo Fisher Scientific). Electron micrographs or tomographic tiltseries were acquired with EPU or tomography software (Thermo Fischer Scientific) at a magnification of 64’000x, corresponding to a physical pixel size of 1.9 Å or 33’000x corresponding to a physical pixel size of 3.7 Å. A nominal defocus range of -2.0 to -4.0 μm was used with a total electron dose of 40 e-/Å2 for 2D imaging or 100 e-/Å2 for tomographic imaging. 3D tomographic reconstructions were performed using the IMOD software.

### Statistical analysis

Statistical analysis was conducted using the Prism9 software. Student t-tests or One-way ANOVA were applied to assess the statistical significance of our results (p-value < 0.05, two-sided). The specific statistical test used in each assay is described in the respective figure legend.

## Results

### Identification of families with severe neonatal encephalopathy and brain malformations

**Fig. 1:**
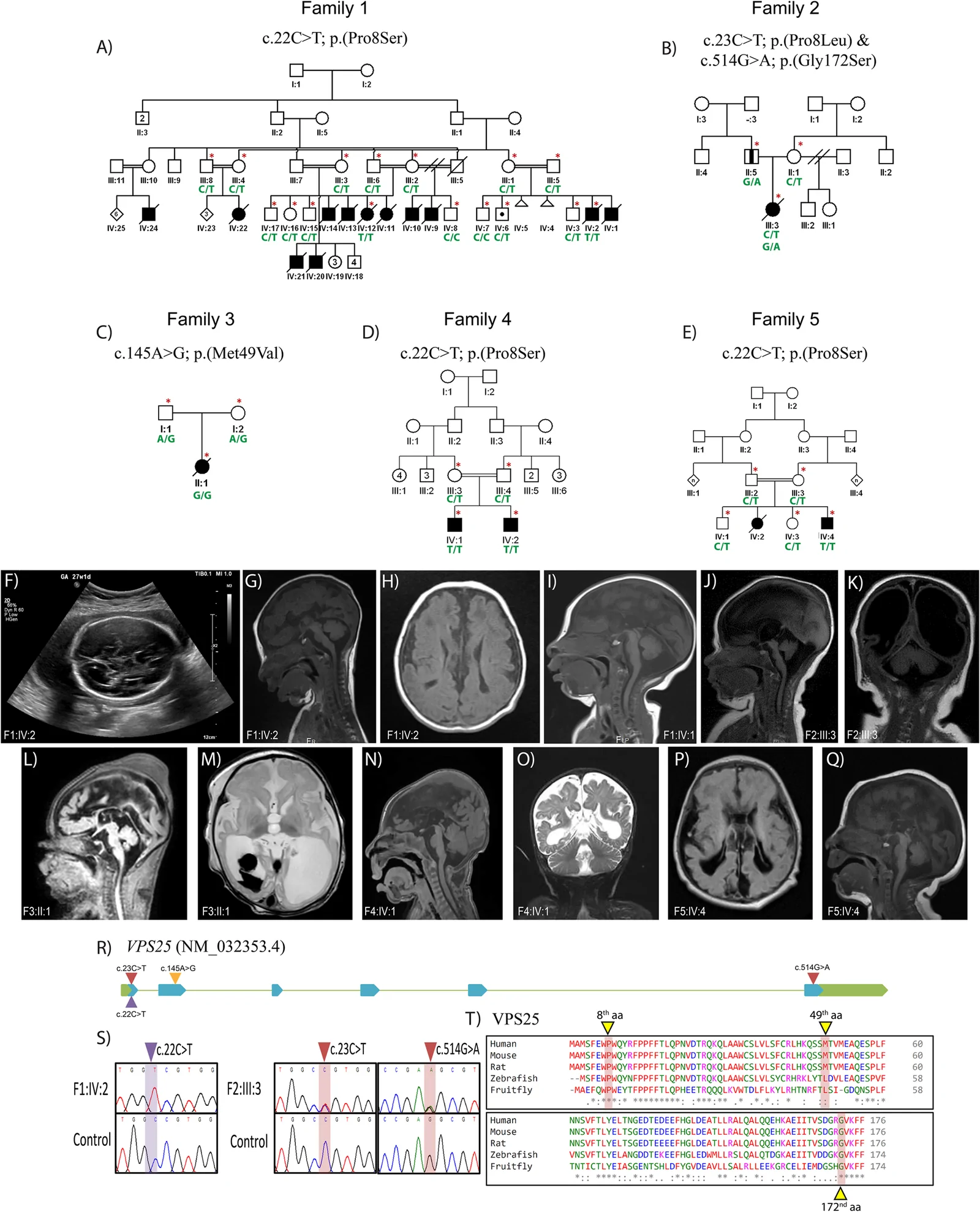
Pedigree, clinical, and molecular findings of families with damaging variants in *VPS25*. **A**-**E** Pedigrees of the five families described in this study. For each family, the identified *VPS25* variant(s) is indicated above the pedigree, while green alphanumeric labels below individual symbols denote the specific genotype of the variant of interest. Predicted protein changes are shown in parentheses in accordance with Human Genome Variation Society nomenclature. Asterisks denote individuals recruited and tested for segregation analysis. In Family 1:IV:6 dotted square denotes a sibling affected with ADHD, and Family 2:II:5 striped square denotes a father affected with neoplastic bone disease and multiple fractures. **F** Ultrasound imaging of F1:IV:2 at gestational age 25 weeks and 6 days revealed absent corpus callosum (tear-shaped ventricles). **G** T1 sagittal brain MRI of F1:IV:2 shortly after birth confirming the ultrasound findings. **H** Axial section showing the agenesis of the corpus callosum and dilated lateral ventricles (**I**) T1 sagittal brain MRI of deceased brother of F1:IV:2 (F1:IV:1) showing absence of corpus callosum as well as mega cisterna magna. **J**,** K** T1 sagittal, and coronal dorsal brain MRI, respectively, of affected individual from the second family (F2:III:3) showing hydrocephalus with severe ventriculomegaly involving the third and lateral ventricles. **L** Sagittal Brain MRI from the index patient of Family 3, showing agenesis of the corpus callosum. **M** Axial section from the same patient demonstrating marked loss of parieto-occipital parenchymal substance and ventriculomegaly of the posterior horns. **N** Sagittal Brain MRI from individual from Family 4, showing agenesis of the corpus callosum. **O **Coronal section from the same individual highlighting dilated lateral ventricles. **P**,** Q** Brain MRI from IV:4 individual from Family 5 indicating agenesis of the corpus callosum. **R** Schematic representation of *VPS25* gene (RefSeq transcript NM_032353.4) with variants indicated: blue blocks denote exons, purple arrow denotes variant identified in Family 1, 4 & 5, red arrows denote variants identified in Family 2, and yellow arrow denotes variant identified in Family 3. **S** Sequence chromatograms showing the identified disease-associated variants in affected individuals of Family 1 and Family 2 compared to the reference sequence from Control samples. **T** Sequence conservation of the VPS25 protein. Multi-species alignment shows high evolutionary conservation of the residues affected by the amino acid substitutions identified in this study (Pro8, Met49, and Gly172; highlighted in red shading). Sequences are shown for Human through Fruitfly

Family 1 is a multiplex consanguineous Saudi family with multiple affected branches. Overall, affected individuals presented with severe neonatal hypotonia and died within months of life with evidence of brain anomalies. (Fig. 1A & Additional file 2). Ultrasound imaging of patient F1:IV:2 at gestational age 25 weeks and 6 days revealed absent corpus callosum (Fig. 1F). Brain MRI showed agenesis of the corpus callosum (Fig. 1G-H). Brain MRI was performed on the affected brother, which showed agenesis of the corpus callosum, colpocephaly, sub-arachnoid hemorrhage, and simplified gyral pattern with prominent Sylvian fissure (Fig. 1I). Ultimately, both siblings died before one year of age due to respiratory failure and cardiac arrest. The infant (F1:IV:12) had a homozygous missense variant, p.P8S, in the protein encoded by *VPS25*.

Family 2 The proband (F2:III:3) was a 7-day-old female with hydrocephalus, seizures, and respiratory distress (Fig. 1B & Additional file 2). MRI showed severe ventriculomegaly involving the third and lateral ventricles with a normal-sized fourth ventricle, consistent with aqueductal stenosis. In addition, there was concern for either mega cisterna magna or small cerebellum (Fig. 1J & K). At 4 years 6 months, she was admitted to the hospital in respiratory failure with hypoxia. Due to the severity of her medical complications, the family elected to transition to comfort measures, and the patient subsequently died. The infant had compound heterozygous missense variants, p.P8L and p.G172S, in the protein encoded by *VPS25*.

Family 3: The index patient (F3:II:1) was the firstborn child to a 28-year-old mother following a pregnancy complicated by fetal ventriculomegaly diagnosed at 34 weeks of gestation (Fig. 1C & Additional file 2). Postnatal MRI demonstrated agenesis of the corpus callosum, ventriculomegaly of the posterior horns, and marked loss of parieto-occipital parenchymal substance consistent with hypoxic-ischemic injury; the latter was probably due to an unexpected placental abruption following rupture of the membranes (Fig. 1L & M). The patient died at day of life 6 due to complications of severe brain damage and multiple organ failure. Trio-whole-exome sequencing revealed a single variant: a homozygous missense variant, p.M49V, in the protein encoded by *VPS25*.

Family 4: The proband (F4:IV:1) is currently in PICU at 9 months of age with difficulties in feeding, seen by a speech and swallowing therapist, and failure to thrive. (Fig. 1D & Additional file 2). Brain MRI revealed agenesis of the corpus callosum, colpocephaly, ventriculomegaly, and paucity of periventricular white matter (Fig. 1N & O). Exome sequencing revealed the same homozygous missense variant as in Family 1, with a shared haplotype indicative of a founder effect.

Family 5: The proband (F5:IV:4) was a 2-year-old boy with severe epileptic encephalopathy and profound global developmental delay (Fig. 1E & Additional file 2). Brain MRI revealed agenesis of the corpus callosum, abnormal lateral ventricles, and putative white matter injury (Fig. 1P & Q). Exome sequencing revealed the same homozygous missense variant as in Family 1, with a shared haplotype indicative of a founder effect.

A brief overview of the individuals’ clinical hallmarks is provided in Table 1, while detailed information is available in Additional file 2.

**Table 1:**
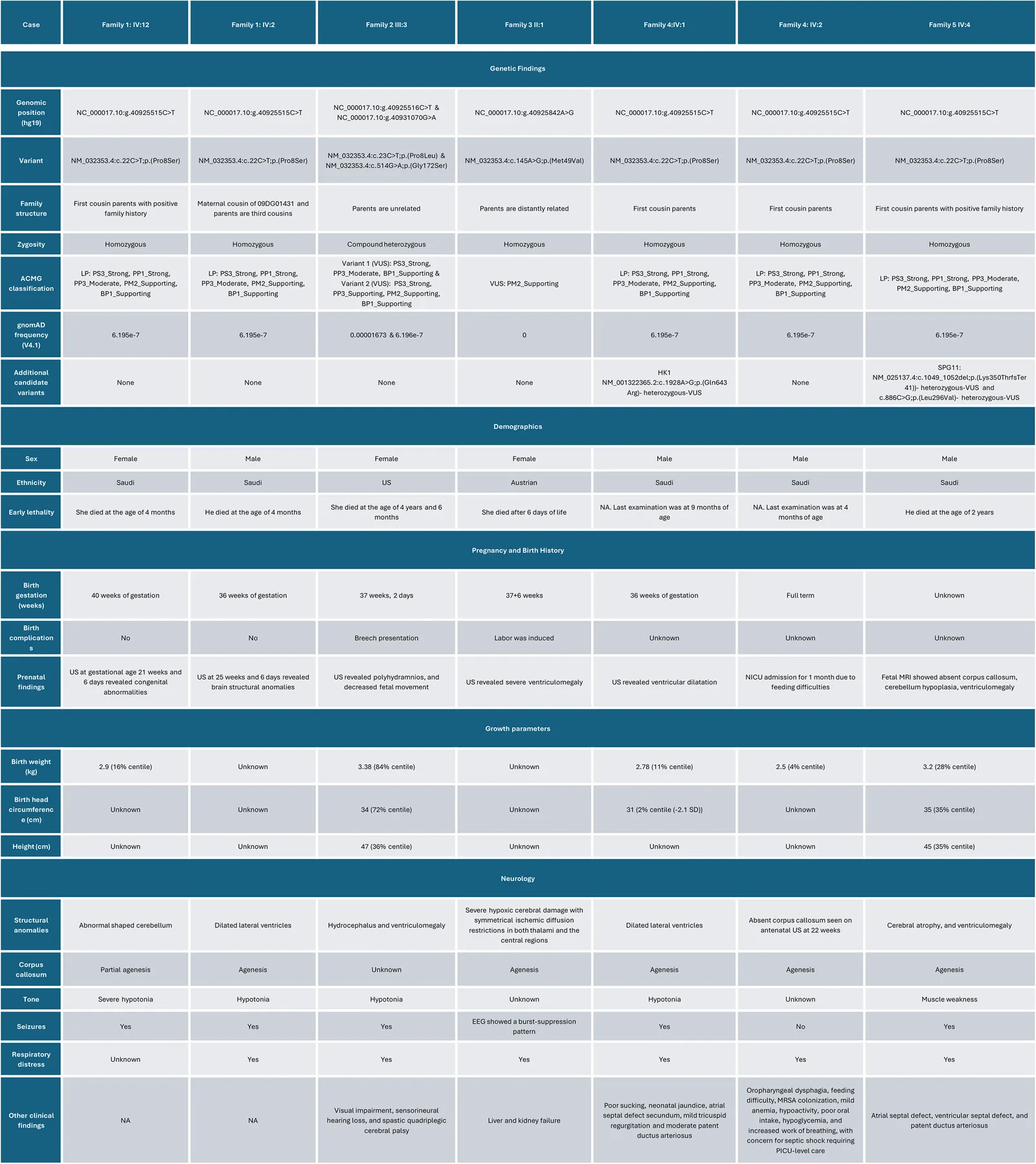
Clinical summary of reported individuals

### Identification of *VPS25* variants in the study cohort

Whole exome sequencing (WES) with autozygosity mapping in Families 1, 4, and 5 revealed a novel homozygous candidate variant *VPS25*, c.22 C > T; p.(P8S), with a shared haplotype indicative of a founder effect (Fig. 1A, D, E, R-T). This was the only candidate variant that fully segregated within the families under the autosomal recessive inheritance model, so it was uploaded to GeneMatcher. This facilitated the identification of Family 2, whose trio WES was non-diagnostic and whose clinical lab uploaded *VPS25* to GeneMatcher as the most compelling candidate based on the identification of compound heterozygous variants: a maternally inherited c.23 C > T; p.(P8L) variant and a paternally inherited c.514G > A; p.(G172S) variant in *VPS25* (Fig. 1B, R-T). It is worth mentioning that residues P8, M49, and G172 are well conserved across species (Fig. 1T). GeneMatcher also facilitated the identification of Family 3 in whom exome sequencing revealed homozygosity for p.(M49V) missense variant in *VPS25*, which was similarly conserved across species.

### Brain abnormalities of *Vps25* hypomorphic mutant mouse embryos

To further investigate the brain anomalies, a mouse model was used [7, 20]. Coronal sections of heads from E14.5 *Vps25* constitutive hypomorphic mouse embryos [7, 20] (*Vps25*^*ENU/ENU*^; *N* = 3) show strikingly dilated lateral ventricles with thinned cerebral cortex. In addition, the thalamus is markedly hypoplastic with an enlarged third ventricle as compared to wild-type controls. Overall, the head is noticeably smaller in mutant embryos (Additional file 1: Fig. S1).

### Halt of ESCRT-II molecular dynamic behavior caused by P8S, P8L, M49V and G172S

**Fig. 2:**
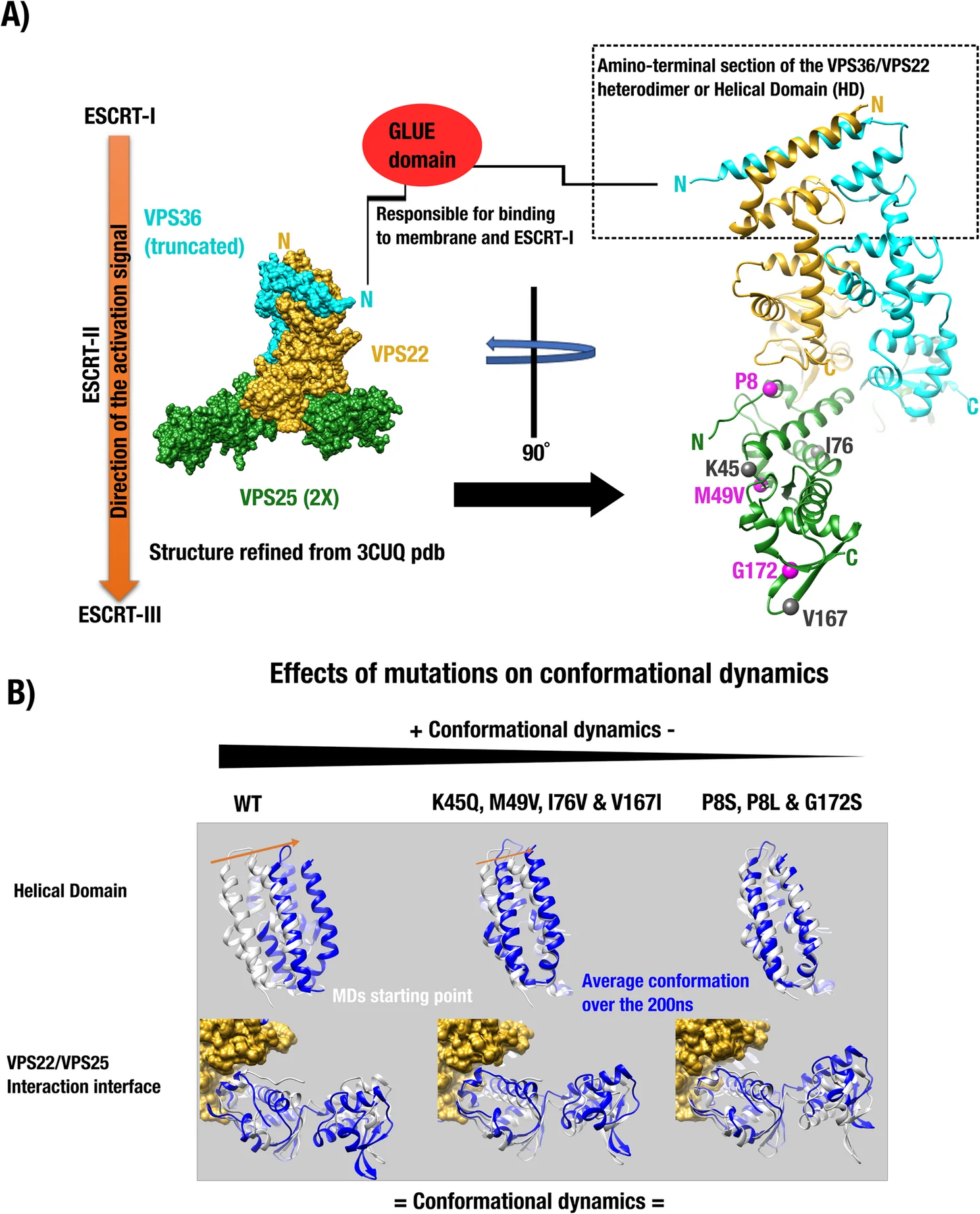
Effects of VPS25 mutations on the molecular dynamics of the Helical Domain of the VPS22/VPS36 section of the ESCRT-II complex.** A** The connectivity of the ESCRT systems and mutations in VPS25 affect the molecular behavior of the ESCRT-II complex during MDs. The tetramer is shown with gold, cyan, and green surfaces for VPS22, VPS36, and VPS25. The lack of the GLUE domain in VPS36 in the refined complex model (3CUQ) is highlighted. **B** Effects of mutations on the conformational dynamics of the ESCRT-II complex are compared. Models represent conformational changes experienced by these complexes under mutagenesis. Results are grouped according to dynamic behavior. Despite the effects caused by VPS25 mutations on HD, no particular change in the dynamic behavior of VPS25 interacting with VPS22 is shown. In light grey and blue are shown the initial state and average conformation maintained during the MDs

In our investigation, we analyzed the evolutionary conservation scores of the variants located at the P8, M49, and G172 positions within the VPS25 protein. While P8 and G172 residues exhibit the utmost level of conservation score the methionine at 49 is moderately conserved (Consurf-database) [40]. The substitutions P8S/L and G172S represent notable alterations to the amino acid side chains. The former introduces a polar or bulky substitution (S or L, respectively) which could potentially impact the molecular “rigidity” typically conferred by proline, while the latter might constrain the “flexibility” associated with glycine [47, 48]. The substitution of methionine 49 by a valine is not as extreme as the examples observed for positions 8 and 172. Subsequently, we evaluated whether these individual mutations could impede the formation of the ESCRT-II complex through in*-silico* experiments. To accomplish this, we refined the truncated form of the ESCRT-II complex derived from the Protein Data Bank (3CUQ). This protein model lacks the amino-terminal segment, also known as the Helical Domain or HD, of VPS36, as well as the GLUE protein domain responsible for membrane binding, ubiquitinated cargo binding, and the establishment of protein-protein interactions with the ESCRT-I system [21, 49, 50] (Fig. 2A). After refining the ESCRT-II truncation via molecular dynamics simulations (MDs), the resultant model served as a template for generating the mutant variants at positions P8, M49, and G172. The assessment of the binding energy of the mutations on VPS25 suggested that these mutations are unlikely to disrupt or impede the formation of the complex. All four mutations (positions 8, 49, and 172) led to a reduction in theoretical binding energies ranging from 5 to 8%, depending on which mutation was present. The theoretical energy of binding between VPS25 and VPS36/VPS22 was calculated to be -346 kJ/mol for the wild type and ≈ -317 kJ/mol for the P8S/L mutants, while M49V exhibited − 319.4 kJ/mol.

We conducted Steered Molecular Dynamics (SMDs) simulations to ascertain whether such a decrease in theoretical binding energy would potentially disrupt the complex, or at least the binding of VPS25. The results indicated that despite the decrease in the energy of binding induced by the mutations, none of the four substitutions would significantly impact the energy required to separate VPS25 from the remainder of the ESCRT-II complex (Additional file 1: Fig. S2).

Then we investigated via MDs if mutations over VPS25 could impact the biological functions of the ESCRT-II complex by altering its molecular dynamics. The MD results indicate that there are no substantial alterations in the molecular behavior of VPS25. Throughout the 200ns simulations, none of the mutants exhibited a significant impact on the average conformation of VPS25 within that time frame (see Fig. 2B). However, a notable disparity was observed at HD section of the VPS22/VPS36 dimer when comparing the molecular dynamics of wild type (WT) VPS25 and the mutant variants P8L, P8S, M49V and G172S. Such section is crucial for bridging the VPS22/VPS36 dimer with the GLUE domain, which VPS36 utilizes to interact with the ESCRT-I system [21]. The GLUE domain was not present in the experimentally derived protein complex models, and it was similarly absent in our refined model (Fig. 2).

The most interesting result from the MDs was that the HD section of the VPS22/VPS36 dimer may be undergoing arrest or reduction in its conformational dynamics by mutations located in different components of the ESCRT-II complex (Fig. 2B). All mutant complexes tested by MDs experiments exhibited a connection between mutations on the VPS25 protein and modulation on the dynamics of the HD of the VPS22/VPS36 dimer. But the most notable predicted changes were only observed for the P8S, P8L and G172S mutations (deleterious), with almost complete obliteration of the molecular dynamics shown by the ESCRT-II WT complex this section of the VPS22/VPS36 protein dimer (Fig. 2B, right side). The MD suggested that this could be caused by secondary structure changes observed at the VPS22/VPS36 dimer interface (Additional file 1: Fig. S3). Consistent with our MDs results, AlphaMissense predicts each of the three missense variants to be highly deleterious [51].

We considered worth repeating that this segment has been acknowledged as remarkably dynamic and is thought to actively bridge the GLUE domain to the remainder of the ESCRT-II core [21]. ESCRT-II specifically targets endosomal membranes by associating with ubiquitinated cargos and engages with the other ESCRT systems through a series of interdependent interactions (Fig. 2A). Given the importance of the GLUE domain for the activity of ESCRT-II, the described VPS25 mutations could at least in part disrupt ESCRT functionality resulting in the observed phenotypes [52, 53].

### The ESCRT-II complex comprising the VPS25 mutants elutes at a higher molecular weight in size exclusion chromatography assays

**Fig. 3:**
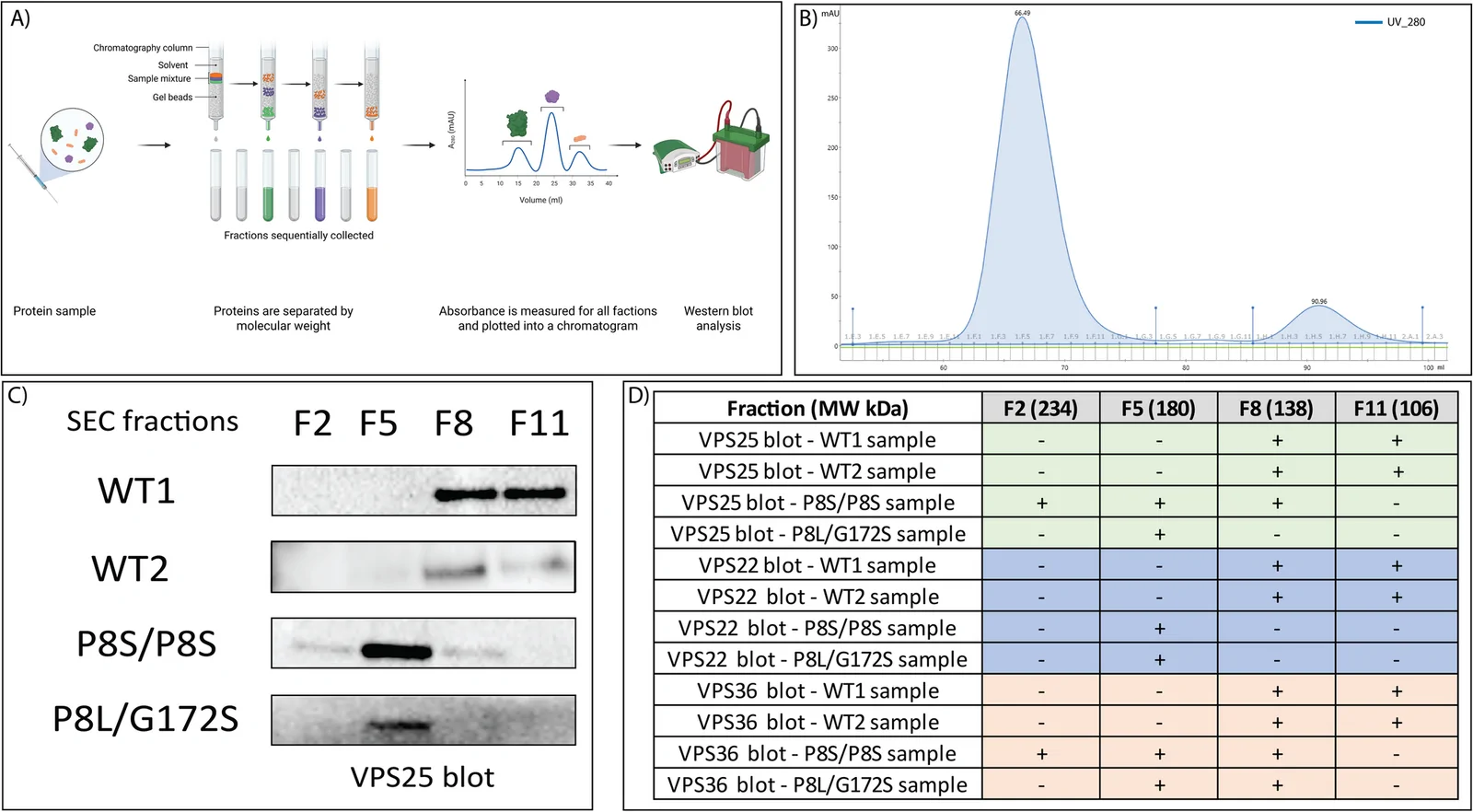
VPS25 mutant ESCRT-II complexes were eluted at higher molecular weight compared to the VPS25 wild-type ESCRT-II complexes.** A** Cartoon illustrating the experimental workflow. Refer to Methods for more details. Figure created by BioRender.com. **B** Representative chromatogram of size exclusion chromatography experiment of the ESCRT-II complex proteins VPS25, VPS36, VPS22, after overexpression and purification in Sf9 insect cells. Wild type and mutant complexes had similar elution profiles (Additional file 1: Fig. S4A and B). The ESCRT-II complex was eluted at the F2-F11 fractions. **C**, **D** Size exclusion chromatography from patient-derived cells. **C** Resulted fractions (F2, F5, F8 and F11) from size exclusion chromatography from patient-derived samples used for western blot to examine the presence or absence of the proteins in the ESCRT-II complex. VPS25 blots are shown here. Blots for VPS22 and VPS36 are shown in Additional file 1: Fig. S6. **D** Based on the western blot results from each protein from the ESCRT-II complex, this table was created. “+” protein was identified; “-“ protein was not identified. WT1 and WT2 samples correspond to cells derived from healthy donors (more details in Methods section), while P8S/P8S refers to cells from IV:12 patient from Family 1 and P8L/G172S refers to cells from III:3 patient from Family 2, both described in the Supplementary Clinical Data document

We next evaluated the effect of the mutant VPS25 on the formation of the ESCRT II complex using Size Exclusion Chromatography assay (Fig. 3A). ESCRT II wild-type (VPS25^WT^) and ESCRT II mutant (VPS25^P8S^ or VPS25^M49V^) were expressed recombinantly in Sf9 insect cells as described in the Methods section. The complexes displayed similar purification profiles (Fig. 3B), including the final step of size exclusion column (Additional file 1: Fig. S4A & B). Furthermore, the relative intensity of VPS25:VPS22:VPS36 in the eluted peaks from the size exclusion column are consistent with the established heterotetrametric stoichiometry of 2:1:1, respectively (Additional file 1: Fig. S4C, D & E) [2] and they were confirmed by Mass Spectrometry (data not shown).

Total lysates of patient-derived cells from two samples of VPS25^WT^, VPS25^P8S/P8S^, and VPS25^P8L/G172S^ cells displayed similar profiles using the size exclusion chromatography assay (Additional file 1: Fig. S5). The same fractions (F2-F11), in which the ESCRT-II complex was eluted during purification from insect cells (Fig. 3B), were analyzed to examine the presence or absence of the corresponding proteins in the size exclusion chromatography fractions derived from patient cell lysates. Interestingly, VPS25 was detected in different fractions between the two wild type samples and the two mutant ones (Fig. 3C). VPS25 was detected in fractions F8 and F11, while in the two mutant samples, VPS25 was detected mainly in fraction F5. The same pattern was observed for the other two proteins that make up the ESCRT-II complex, VPS22 and VPS36 (Fig. 3D, Additional file 1: Fig. S6). In order to determine the average molecular weight of the complexes in each fraction, we used an approach based on calibration parameters developed in a previous study [54]. This calculation revealed that the average difference in size of the complex containing the wild-type VPS25 compared with the complex containing the VPS25 mutants was about 40 kDa. Indeed, the complex comprised of the VPS25^WT^ protein eluted around 138 kDa, which is within the normal range, while the complex comprised of mutated VPS25 eluted around 180 kDa. These results suggest that the complex with the mutated VPS25 comprises additional proteins that are not found in the wild type complex.

### VPS25 mutants co-immunoprecipitate distinctive sets of proteins

**Fig. 4:**
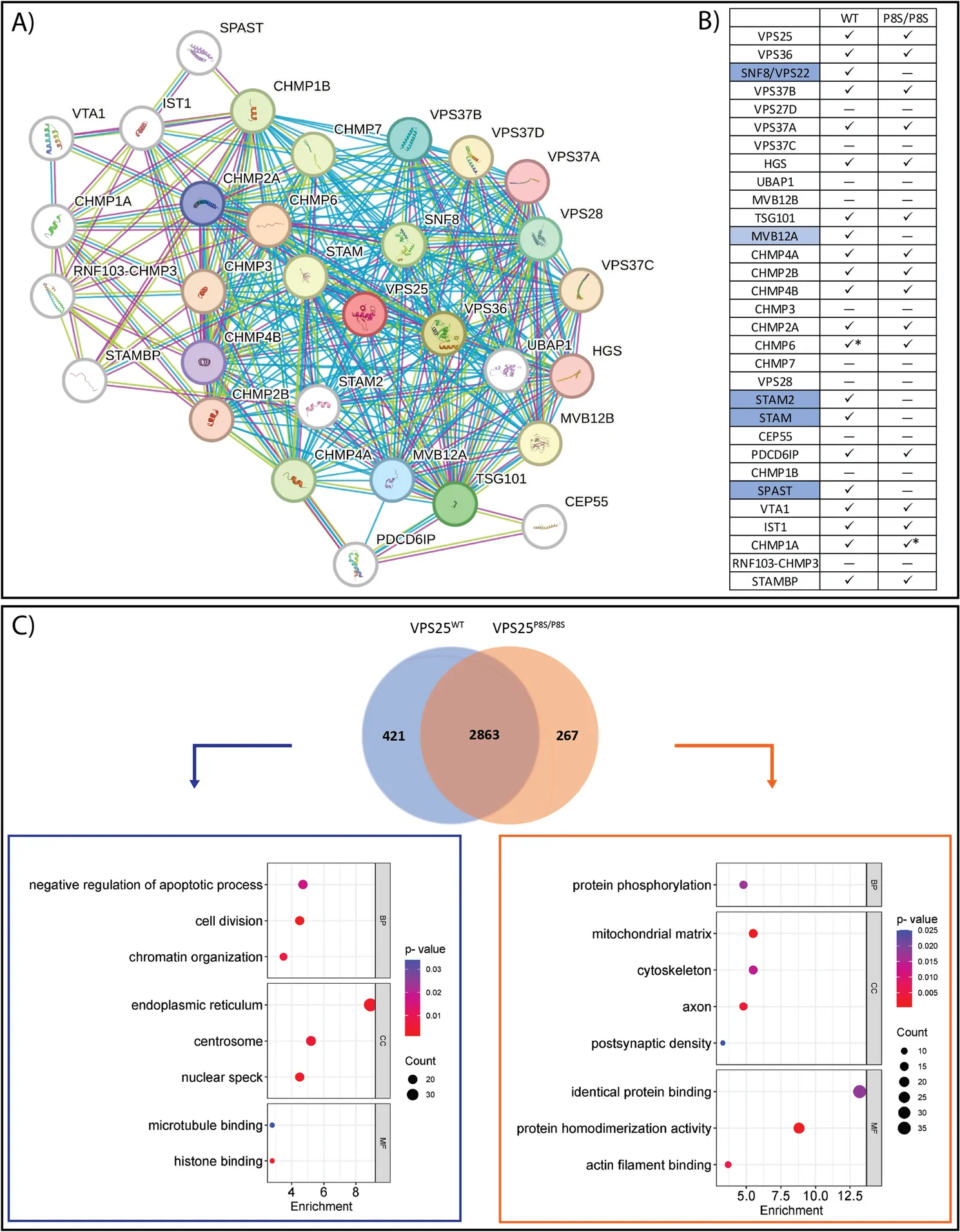
Mass Spectrometry analysis of Co-Immunoprecipitation studies targeting VPS25 revealed that distinctive sets of proteins associate with mutant and wild-type versions, suggesting different functional consequences.** A** Top 30 interacting proteins with VPS25 according to the STRING online tool. **B** From these 30 proteins, five were identified only in the VPS25^WT^ sample (highlighted in blue). To identify the proteins from the pull-down, two independent software with different algorithms were used (refer to Methods). “✓”protein was identified, “—” protein was not identified, “*” protein was identified using only one of the software used. Two independent co-immunoprecipitation experiments followed by qualitative mass spectrometry were performed. **C** Proteins identified using both software algorithms, were used to create this Venn diagram. 2863 proteins were identified in both pull-downs, 421 only in the wild type and 267 only in the homozygous P8S/P8S sample. Gene Ontology, including Biological Process (BP), Cellular Compartment (CC), and Molecular Function (MF) analysis was performed. Some of the unique categories enriched in each sample with p-value < 0.05 are shown in the dotplots

To further investigate the complex formation of ESCRT-II and to identify the putative changes of its interactome in the VPS25 mutant samples of ESCRT-II, co-Immunoprecipitation of VPS25 (Additional file 1: Fig. S7), followed by mass spectrometry, was performed on total cell lysates of *VPS25*^WT^ and *VPS25*^P8S/P8S^ samples. First, using the String online software [43], we identified the top 30 direct or indirect interacting proteins of VPS25 (Fig. 4A). Five of them (Fig. 4B, highlighted blue), were identified in the wild-type Co-IP elution product, but not in the mutant one: Snf8 or VPS22, the Signal transduction adaptor molecule 1&2 (STAM and STAM2), the Multivesicular body subunit 12 A (MVB12A) and the spastin. Since VPS22 is an essential protein for the integrity of the ESCRT-II complex, its absence in the VPS25mut Co-IP indicates a potentially defective complex. Apart from VPS22, mutated VPS25 did not co-precipitate proteins that are part of other ESCRT complexes. These findings are consistent with our in silico interaction data, which demonstrated that the mutated ESCRT-II complex is unable to associate with other ESCRT family members. Together, these results indicate that the mutation disrupts the physical assembly and coordination between ESCRT subcomplexes. More specifically, STAM proteins, in cooperation with the hepatocyte growth factor-regulated tyrosine kinase substrate (HGS), form the ESCRT-0 complex [55], which mediates the recognition and transport of ubiquitinated cargo towards lysosomal degradation pathways [56]. This suggests that the protein recycling system might be hampered. On the other hand, MVB12A is part of ESCRT-I complex [57]. Loss of function of MVB12 alters the cargo selection of MVBs resulting in alterations in the proteins loaded into the MVBs [57] and most likely to their engulfed exosomes. Furthermore, spastin is an important interacting protein of ESCRT-III complex mediating regulation of polarized membrane trafficking [58].

We sought to better understand the properties of the interactome resulting from the VPS25^P8S/P8S^ compared with the VPS25^WT^ by performing a Gene ontology (GO) analysis (refer to Methods) of the proteins that were pulled down. More specifically, 421 proteins were identified interacting only with the wild type VPS25, while 267 proteins were identified interacting exclusively with the VPS25^P8S/P8S^ (Fig. 4C, top). Using the DAVID online tool [44, 45], GO categories unique to each sample were revealed (Fig. 4C, bottom). For example, GO analysis of cellular compartments, revealed 21 proteins related to centrosomes in the VPS25^WT^ pull-down, while this category was not found in the analysis of the VPS25^P8S/P8S^ pull-down. On the other hand, proteins related to neuronal axons and postsynaptic density were identified with the VPS25^P8S/P8S^ but not with the VPS25^WT^ sample. Interestingly, the GO-molecular function analysis also revealed that there was enrichment in microtubule binding proteins with the VPS25^WT^ pull-down, while there was enrichment in actin filament binding proteins with the VPS25^P8S/P8S^ pull-down. Indeed, abnormal morphology of actin filaments was observed in mutant cells under fluorescence microscopy (Additional file 1: Fig. S8).

### Electron microscopy studies illustrate severe abnormalities in MVBs and lysosomes in *VPS25* mutant cells compared to control

**Fig. 5:**
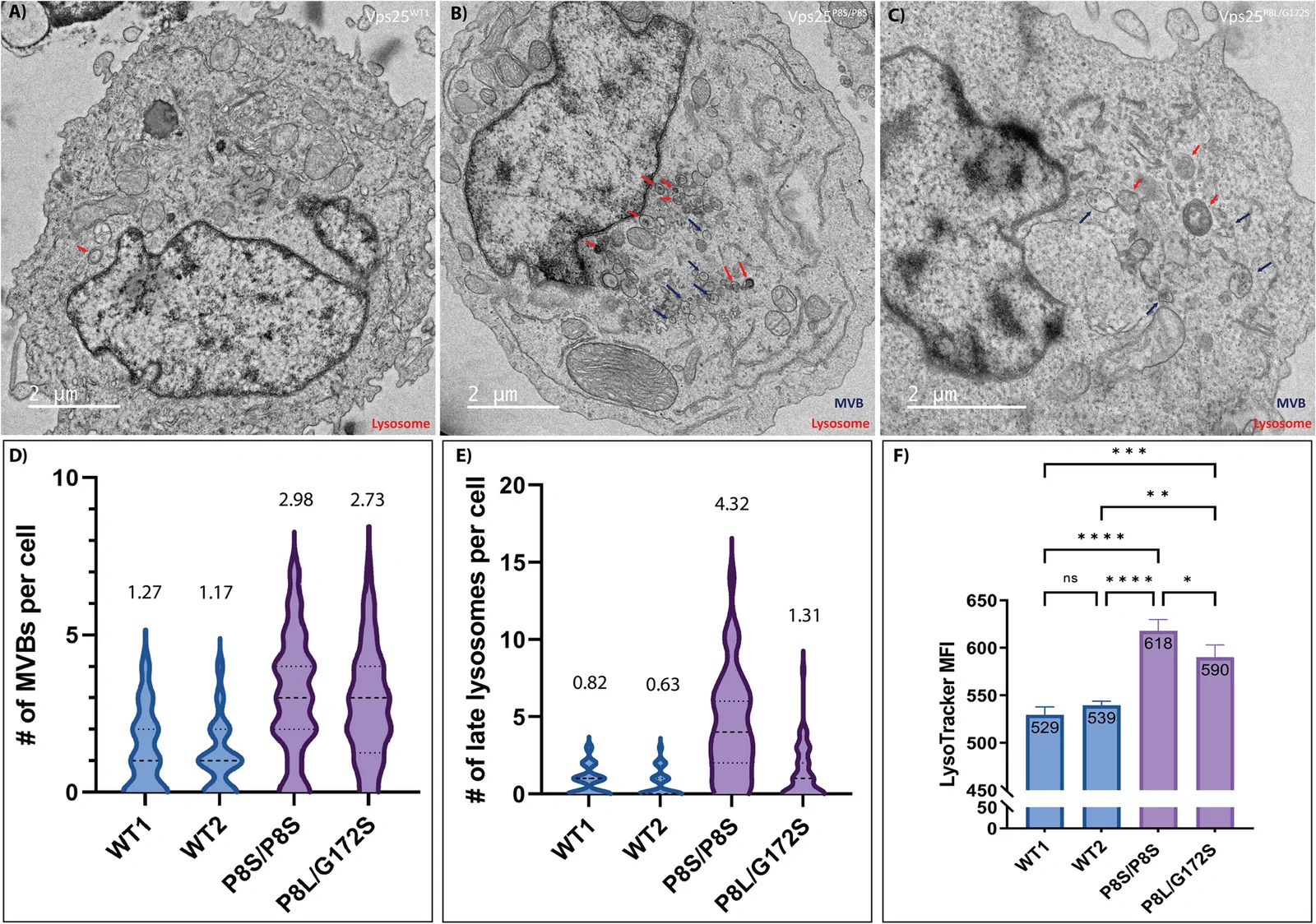
Cells with mutation in VPS25 display abnormal increased number of MVBs and lysosomes. **A**-**C** TEM imaging of wild type (**A**),VPS25P8S/P8S (**B**) and VPS25P8L/G172S (**C**) mutant cells showing increased numbers of MVBs and lysosomes. Blue arrows indicate MVBs and red arrows indicate lysosomes. **D** Quantification of MVBs per cell from at least 40 imaged cells from each group. The mean value of each sample is shown above each violin plot. One-way ANOVA statistical test displayed a p-value < 0.0001. **E** Quantification data of late lysosomes per cells. The mean value of each sample is shown above each violin plot. One way ANOVA statistical test displayed a p-value < 0.0001. **F** Flow cytometry analysis using LysoTracker dye was used to quantify the lysosome signal in healthy VPS25^WT^ cells (blue) and VPS25^mut^ cells (purple). Data are averaged from *n* = 3 independent experiments. One-way ANOVA with Tukey HSD test was performed; (*p*-values: *<0.05, **<0.01, ***<0.001, ****<0.0001)

Comparison of TEM images between wild type lymphoblasts and lymphoblasts derived from patients revealed increased abundance of MVBs in the mutant cells (Fig. 5A-C), consistent with previous work in mice [7]. Furthermore, quantification data of the number of MVBs per cells (Fig. 5D) revealed that lymphoblasts carrying mutation in VPS25 displayed more than double the amount of MVBs per cell, compared to the wild type lymphoblasts. Furthermore, TEM imaging of VPS25^mut^ lymphoblasts also revealed increased abundance of lysosomes compared to controls, consistent with previous work in mouse embryonic fibroblasts that mutations in VPS25 could lead to abnormalities of lysosomal processes [7] (Fig. 5A-C). Quantification of the number of late lysosomes per cell (Fig. 5E) revealed that lymphoblasts carrying mutations in VPS25 displayed an increased number of late lysosomes per cell, compared to the wild-type lymphoblasts. To further validate these findings, flow cytometry was performed to detect lysosomes using the LysoTracker dye (Fig. 5F). In accordance with our previous data, healthy cells had a significantly lower signal of lysosomes compared to the VPS25 mutated cells, especially VPS25^P8S/P8S^.

### *VPS25* mutant cells display fewer secreted sEVs and altered sEV protein cargo

Exosome biogenesis is mediated through the inward budding of the membrane of early endosomes, which subsequently develop into MVBs [9]. This process has two distinct pathways, the ESCRT-dependent and the ESCRT-independent pathway [9–11]. According to our simulation (Fig. 2) and Co-IP data (Fig. 4), the disease-associated variants in the VPS25 protein appear to cause abnormal interactions between ESCRT-II family with other ESCRT complexes. Therefore, we hypothesized that the ESCRT-dependent exosome biogenesis pathway may be defective, akin to how the ESCRT inhibitor, manumycin A, partially inhibits exosome biogenesis [11, 12].

**Fig. 6:**
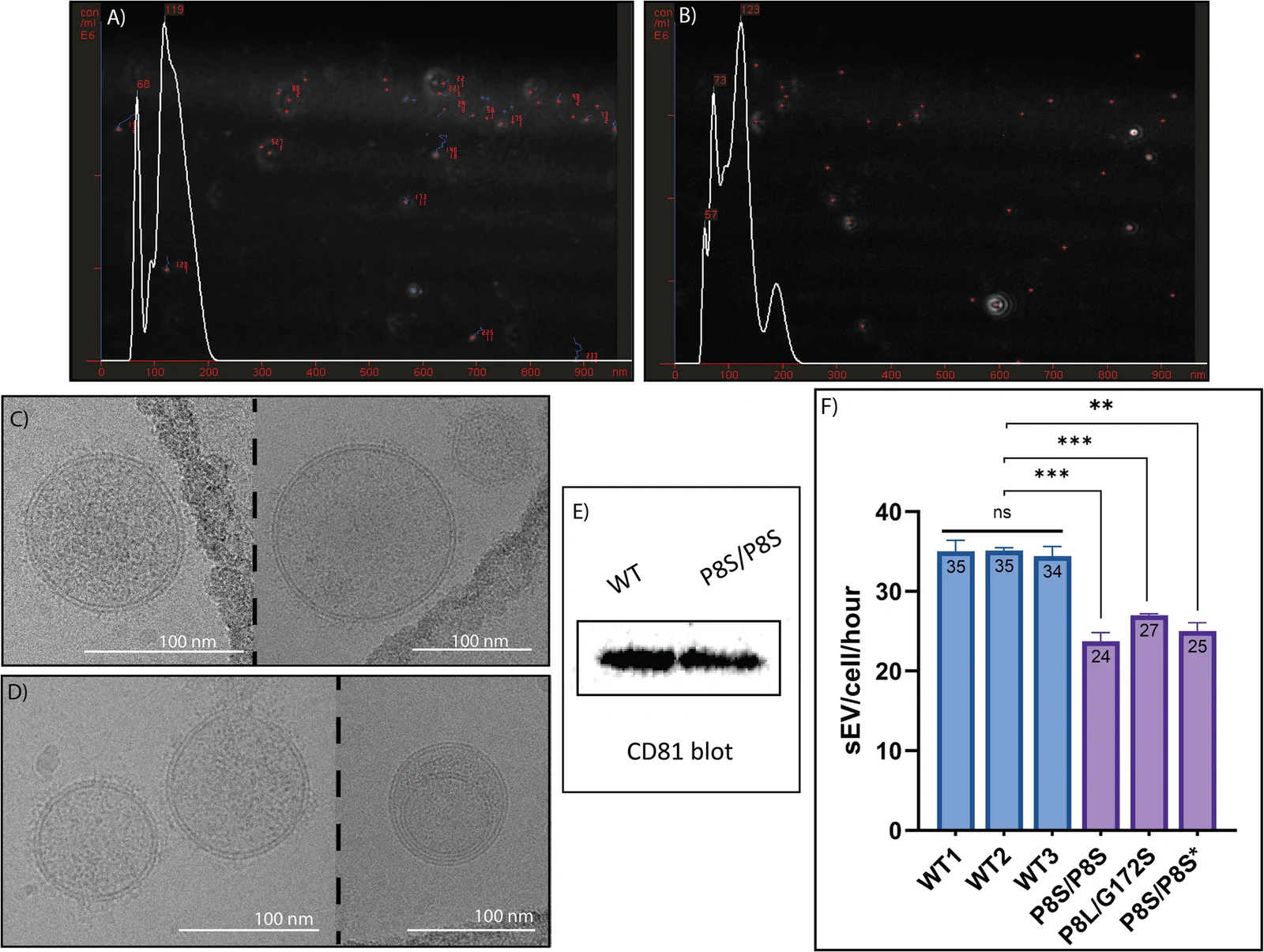
Mutation in VPS25 decreased the number of secreted sEVs. **A**, **B **Nanoparticle tracking analysis (NTA) showing the size distribution of the isolated sEVs derived from wild-type (**A**) and *VPS25* mutant (**B**) cells. **C**, **D** Cryo-electron microscopy (cryo-EM) images of the isolated sEVs derived from wild-type (**C**) and mutant (**D**) cells. Scale bar = 100 nm. **E** Western blot of the sEV marker CD81 from total lysates of isolated sEVs derived from *VPS25*^WT^ and *VPS25*^P8S/P8S^ cells. **F** Quantification of the sEVs released using the NTA machine. sEVs were quantified as the number of sEVs, released per cell per hour. WT1-3 corresponds to sEVs released by cells derived from cells from healthy patients. P8S/P8S and P8L/G172S correspond to sEVs released by cells carrying the *VPS25*^P8S/P8S^ and *VPS25*^P8L/G172S^ mutations, respectively. P8S/P8S* corresponds to the cells-derived from individual from Family 4. Data are averaged from *n* = 3 independent experiments. Asterisks above the chart bars indicate the highest p-values obtained from student’s t-tests comparing each mutant sample to the wild-types, *p*-value ** < 0.01, *** < 0.001

Previous work in our lab illustrates that we can efficiently isolate and characterize sEVs from cell lines as well as primary hematopoietic cells [42]. To this end, sEVs were isolated from lymphoblasts derived from normal individuals and *VPS25* mutant patients using a serial ultracentrifugation method [42] and their size distribution was calculated using a Nanoparticle Tracking Analysis system [42] (Fig. 6A & B). sEVs at the 100k x g pellet displayed a normal size distribution between a 50–200 nm diameter and had a typical intraluminal organization as observed using Cryo-EM (Fig. 6C & D). Moreover, the sEVs displayed the classical sEV markers, such as CD81 (Fig. 6E, Additional file 1: Fig. S9). Interestingly, more complicated structures were observed in a small portion of the sEV (Additional file 1: Fig. S10A). The percentage of sEVs displaying more complicated structures was increased in the VPS25^P8S/P8S^ patient compared with the wild type samples (Additional file 1: Fig. S10B). Interestingly, such structures have also been observed in Cryo-EM images of sEVs derived from the cerebrospinal fluid of Parkinson’s disease patients [59].

After confirming the successful sEV isolation from the lymphoblasts, the sEV release rate was calculated (see Methods for more details) and quantified as the number of sEVs released by a cell per hour. As illustrated in Fig. 6F, sEVs derived from VPS25^WT^ lymphoblasts were significantly higher in number compared with sEVs derived from *VPS25* mutant lymphoblasts. This was corroborated by electron microscopy, which revealed the presence of abnormally higher numbers of MVBs in *VPS25* mutant lymphoblasts compared with control (see Fig. 5D).

**Fig. 7:**
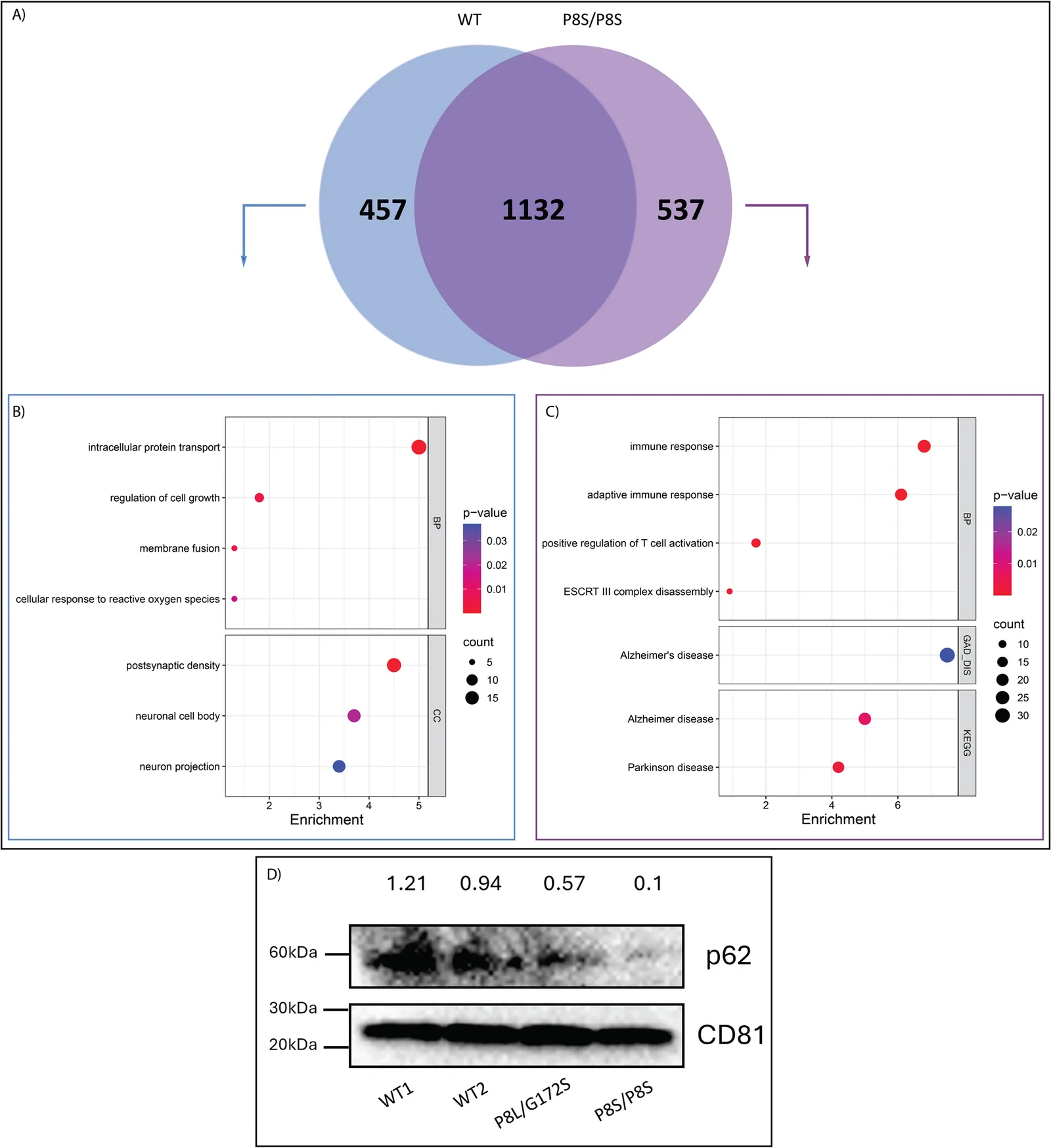
Mutation in VPS25 caused alterations in the sEV protein cargo composition. **A** Protein cargo identified in sEVs derived from wild type (*VPS25*^WT^) and mutated (*VPS25*^P8S/P8S^) lymphoblasts. **B** Unique categories of functional analysis of the proteins identified only in the wild type derived sEVs. **C** Unique categories of functional analysis of the proteins identified only in the patient derived sEVs. BP: Biological Process, CC: Cellular Compartment, MF: Molecular Function, KEGG: Kyoto Encyclopedia of Genes and Genomes, and GAD_DIS: Gene Association Database-Disease. **D** Western blot against p62(SQSTM1) (top) and CD81 (bottom). In each sample, the lysate of 3 × 10^9^ sEVs was used. The numbers above the blots indicate the ratio between the intensity of the signal of p62 blot against the CD81 blot (*n* = 3)

Given that the ESCRT family, apart from the biogenesis and secretion of the exosomes [2, 9], plays a central role in loading cargo into MVB [1, 56, 60] and their subsequently derived exosomes, we sought to investigate whether the disease-associated variants in VPS25 could affect the protein cargo of sEVs. To accomplish this, sEVs were isolated from *VPS25*^WT^ and *VPS25*^P8S/P8S^ lymphoblasts, and high-sensitivity mass spectrometry was performed to reveal their protein cargo. As shown in Fig. 7A, a significant number of proteins differed between these samples. About 32% (537 proteins out of 1669) of the proteins that were found in the sEVs derived from the mutated cells were not found in the sEVs derived from the wild type cells. Similarly, 457 proteins were identified only in the sEVs derived from wild type lymphoblasts. These results indicate that, apart from quantitative differences in the number of sEVs released, the disease-associated variants in *VPS25* cause qualitative differences in the proteome of the sEVs.

Seeking to focus on the distinct proteins identified within the sEVs derived from *VPS25*^WT^ and *VPS25*^P8S/P8S^ cells respectively, we revealed interesting unique functional categories enriched in each sample. For example, proteins identified from the *VPS25*^WT^ derived sEVs, showed enrichment in GO categories related to neurons such as “postsynaptic density” and “neuronal cell body” (Fig. 7B), while such proteins were not found in the *VPS25*^P8S/P8S^ derived sEVs samples. A consistent pattern was observed upon analyzing the proteomic cargo of *VPS25*^P8L/G172S^ derived sEVs compared to control sEVs (Additional file 1: Fig. S11). Moreover, proteins that contribute to healthy development and maintenance of neurons [61–63] such as sodium voltage-gated channel α subunit 1 (SCN1A), stathmin 2 (STMN2) and sequestosome 1 (SQSTM1), which were uncovered as part of the *VPS25*^WT^ derived sEVs cargo only. Alternatively, the unique proteomic cargo of the *VPS25*^P8S/P8S^ derived sEVs revealed distinct enrichment in immune response-related categories such as “adaptive immune response” and “positive regulation of T cell activation” (Fig. 7C), suggesting a key role in inflammation. Indeed, in line with this, the results showed an enrichment in categories related to neurodegenerative diseases such as Alzheimer’s disease, which was confirmed by two different databases. To validate the sEVs proteomics data, sEVs lysates were run for western blot to detect SQSTM1 (or p62). As shown in Fig. 7D, there is a dramatic decrease of the amount of p62 present in sEVs derived from *VPS25* mutant cells compared with the wild type controls, confirming our mass spectrometry data.

## Discussion

Intercellular communication is a ubiquitous feature of multicellular organisms and is necessary for homeostasis. In the developing central nervous system, this communication involves the secretion of extracellular vesicles to promote neuronal proliferation and differentiation and modulation of neuronal activity [64]. Recent reports have shown that sEVs play a major role in maintaining and supporting neurons [65, 66]. More specifically, it has been suggested that protein cargo from oligodendrocytes is delivered via their derived sEVs to neuronal axons [65]. Interestingly, oligodendrocyte-derived sEVs, when lacking specific proteins, such as proteolipid protein (PLP), can lead to neuron degeneration [65]. Altered sEVs cargo has been observed in a number of neurodevelopmental disorders, e.g., Rett syndrome, Down syndrome, and fetal alcohol syndrome [67]. However, a causal link between sEV biogenesis and neurodevelopmental disorders has been largely elusive. In this study, we suggest that recessive deleterious variants in *VPS25* lead to a severe and novel neurodevelopmental disorder likely mediated by abnormal sEV biogenesis. Our analysis of five families with this neonatal encephalopathy revealed that each segregates a likely deleterious biallelic variant in *VPS25* with associated significant impact on the number of sEV and the nature of their protein cargo.

The clinical phenotype associated with biallelic *VPS25* variants was remarkably consistent across the five unrelated families. Affected individuals presented with early-onset neurological symptoms, including neonatal hypotonia, seizures, and respiratory difficulties, as well as profound developmental delay. Brain imaging consistently revealed corpus callosum abnormalities—including complete agenesis—along with ventriculomegaly and other structural malformations, some of which were detectable prenatally. Electroencephalographic findings were consistent with epileptic encephalopathy. These findings strongly support a role for VPS25 in early human brain development. Indeed, observations from coronal sections in *Vps25* hypomorphic mouse embryos, such as ventricular enlargement and reduced cortical thickness, further reinforce the contribution of VPS25 dysfunction to a neurodevelopmental disorder. It is noteworthy that the Vps25 hypomorphic mouse model exhibited phenotypic features not observed in human patients, a discrepancy potentially attributable to the ~ 90% reduction in protein levels in the mouse model compared to the relatively normal protein expression observed in the human patients. Similar neurodevelopmental phenotypes have also been described for variants in *SNF8* (*VPS22*), another ESCRT-II subunit, although with a broader clinical spectrum that includes both severe encephalopathy and milder features such as optic atrophy and ataxia [4]. This supports the critical role of ESCRT-II integrity in early brain development and highlights potential subunit-specific contributions to disease severity.

The multiple interactions in ESCRT systems with ubiquitinated cargo, as well as with membranes, safeguard an efficient chain lipid-sorting machinery that ultimately results in the formation of MVBs [53]. In the case of the lipid-binding activities of the ESCRT-II system, both the GLUE domain of VPS36 and the first helix of VPS22 are involved. Based on our simulation results, this first alpha helix is part of the helical domain, the section of the protein complex most affected by mutations in the VPS25 component of ESCRT-II. It is therefore easy to imagine that disruption of this well-balanced functional framework could have an impact on the biological function of the ESCRT-II complex [21]. The results of our wild-type simulations agree with those reported by Jun Im & Hurley [21], illustrating that the helical domain is the most dynamic section in WT simulations, having an intrinsically high mobility. These results need further experimental validation but suggest allosteric dysregulation by mutations in the VPS25 protein members of the ESCRT-II system.

The size exclusion chromatography experiments performed on the wildtype and mutant *VPS25* cells revealed that the VPS25 mutant proteins eluted in the same fractions as the other ESCRT-II complex proteins, suggesting that the complex itself is not disrupted. Interestingly, however, the VPS25 mutant ESCRT-II complex was eluted at a higher molecular weight compared with the VPS25 wild-type complex. This suggests that new abnormal interactions may be occurring. However, VPS22 was not identified in the high-sensitivity mass spectrometry analysis performed on the VPS25 Co-IP sample from mutant cells, while it was present in the Co-IP sample from the wild type cells. We suspect this could, in principle, be due to the fact that during Co-IP experiments, there are technically more washing steps, which may not preserve the weaker interactions as compared with the size exclusion chromatography experiments, where weaker interactions are better conserved [68]. Also, in the case of VPS22, the mutant VPS25 could cause the interaction of VPS22 with the rest of the ESCRT-II complex to become less stable. Overall, the Co-IP Mass Spectrometry data indicate that the mutant VPS25 causes fewer interactions between the ESCRT-II complex and the other ESCRT family members, which might lead to “communication problems”. Proper interaction among the ESCRT complexes is vital for their physiological function. For instance, interaction of VPS20 (or CHMP6), a core subunit of the ESCRT-III complex, with ESCRT-II is required for the assembly of the ESCRT-III complex [1, 69], which has a vital role in the formation and abscission of intraluminal endosomal vesicles [70]. Given that ESCRT complexes work as a cascade, delivering cargo from one to another, this decrease in interactions can explain the phenotypic changes observed regarding the lysosomes and exosomes [1, 2]. Furthermore, the mutant VPS25 seemed to initiate interactions of the ESCRT-II complex with abnormal partners. For example, there was enrichment in the GO “actin filament binding” category, among the proteins solely pulled down by the VPS25^P8S/P8S^, while this category was not found in the protein solely pulled down by the VPS25^WT^. Interestingly, the actin filaments in the cells where *VPS25* was mutated seemed to have a more irregular shape and direction.

Consistent with previous work in mice [7], mutations in *VPS25* led to increased numbers of MVBs and lysosomes observed using electron microscopy and by measuring lysosome signals via flow cytometry. Both patients with VPS25 mutations had more than twice the number of MVBs compared to the control cells. Interestingly, cells with the *VPS25*^P8S/P8S^ variant had a significantly higher number of lysosomes compared to cells with the *VPS25*^P8L/G172S^ variant. When compared to *VPS25*^WT^ cells, it was evident that cells with the *VPS25*^P8S/P8S^ variant exhibited defects that were more pronounced than those with the *VPS25*^P8L/G172S^ variant. This observation is in line with the phenotypic characteristics and life duration of the patients.

sEV biogenesis and secretion is a multistep process, involving the function of ESCRT family complexes [9, 10]. Dysregulation of the ESCRT chain pathway can lead to a reduction in sEVs production [12, 13]. Indeed, the VPS25 variants caused an increase of MVBs inside the patient cells and the subsequent decrease in the number of sEVs, they release. The cells carrying the *VPS25*^P8S/P8S^ and *VPS25*^P8L/G172S^ variants released significantly lower numbers of sEVs compared with the wild type ones. Surprisingly, the sEVs quantification data revealed the same pattern for the patients’ cells, as for the lysosomes semi-quantification. Additionally, a very interesting observation was made by examining the sEVs under Cryo-EM, where sEVs derived from *VPS25*^P8S/P8S^ cells displayed more complex-multilayer structures. These types of structures have been reported in many diseases and have just started to be studied [71, 72].

Additionally, differences within the proteomic cargo of sEVs derived from wild type cells and those derived from mutant cells of patients may help in understanding the molecular causes of this form of encephalopathy. For instance, SCN1A is a voltage-gated sodium channel initiating action potentials in neurons [73]. This protein was found in the wild-type derived sEVs but not in mutant sEVs. Mutations in SCN1A have been linked to a range of genetic epileptic syndromes [73]. About 80% of these mutations are loss of function mutations that cause severe early infantile epileptic and developmental encephalopathy (Dravet’s Syndrome) [73, 74]. Another stark example of proteins identified only in the wild type but not in the mutant-derived sEVs is SQSTM1, a protein classified within the “cellular response to reactive oxygen species” category of proteins [75]. Interestingly, recent work has highlighted its important role in maintaining homeostasis in the neurons, as loss of function of SQSTM1 leads to neurodegenerative diseases [63]. Intriguingly, recent studies have explored the importance of protein cargo received from oligodendrocyte-derived sEVs to axon health [66]. Given that ESCRT family complexes are involved in the sorting of proteins into vesicles [1, 56, 60], the differential presence of sEV proteins in mutant versus wild type sEVs may implicate specific mechanisms of action on axons. These findings are consistent with recent reviews highlighting the central role of ESCRT-II in neurons, in various processes critical for neuronal homeostasis and development, such as endosomal cargo sorting [76].

A limitation of this study is that our observations were conducted in non-neuronal cells, whereas neurons are the primary affected cell type in this disorder. However, given that the *VPS25* mutation affects a conserved and fundamental mechanism present in all human cells, it is reasonable to hypothesize that the reported dysregulations, such as miscommunication among ESCRT family members, and alteration in protein sorting of sEVs, also occur in neurons. Future studies utilizing neuronal samples will be essential to validate this mechanism in the relevant tissue.

## Conclusions

In sum, we report a novel neurodevelopmental disorder characterized by congenital severe encephalopathy with brain developmental defects, epilepsy, and early mortality. We show that this disorder is likely caused by biallelic hypomorphic missense variants in *VPS25*, an essential component of ESCRT-II. The significant and consistent impact that these variants have on sEV biogenesis, such as exosomes, in patient cells suggests a potential mechanism, given the established role of sEVs in CNS development. While future cases will be needed to further delineate the phenotype and mutational spectrum of this condition, the families we report here offer a new take on abnormal sEVs as drivers of neurodevelopmental disorders.

## Supplementary Information

Additional file 1: Supplementary Information. Contains supplementary antibody information (Table S1) and supplementary Figs. S1–S11 supporting the mouse, molecular dynamics, protein purification, microscopy, western blot, Cryo-EM, and sEV proteomics analyses.

Additional file 2: Supplementary Clinical Data. Contains detailed clinical summaries for Families 1–5.

Additional file 3: Full-length images of western blots shown in the main figures. Contains uncropped/full-length blot images corresponding to Figs. 3C, 6E, and 7D.

## Data Availability

All data supporting the findings described in this manuscript are available in the article, its Supplementary data files, and from the corresponding authors upon reasonable request. Requests should be sent to Jasmeen S. Merzaban at jasmeen.merzaban@kaust.edu.sa and Fowzan S. Alkuraya at falkuraya@liferaomics.com.sa. Due to local privacy laws, confidentiality requirements, and the potentially identifiable nature of human genomic data, requests for access to individual-level genomic data are subject to prior approval from the local IRB. The KFSHRC IRB can be contacted at IRB@kfshrc.edu.sa, with an expected response timeframe of approximately two months. Approved data requests will be fulfilled within 30 working days of receipt of all required approvals and, where applicable, completion of a data-use agreement.

## Abbreviations

CO-IP: Co-immunoprecipitation
Cryo-EM: Cryogenic Electron Microscopy
ESCRT: Endosomal Sorting Complex Required for Transport
MDs: Molecular Dynamics (simulations)
MRI: Magnetic Resonance Imaging
MVB: Multivesicular Body
NTA: Nanoparticle Tracking Analysis
sEV: Small Extracellular Vesicle
TEM: Transmission Electron Microscopy
VPS: Vacuolar Protein Sorting
