# Supplementary material for: Recessive variants in ESCRT-II component VPS25 alter vesicular transport and small extracellular vesicle processing in lethal neonatal encephalopathy
Source: Genome Med. 2026 Sep 14;18:165. doi: 10.1186/s13073-026-01693-z (PMC13712088; doi:10.1186/s13073-026-01693-z)
Supplement: Supplementary file 1 [file 13073_2026_1693_MOESM1_ESM.pdf]

Additional file 1 – Supplementary Information

Recessive variants in ESCRT-II component VPS25 alter vesicular transport and small extracellular vesicle processing in lethal neonatal encephalopathy

Ioannis Isaioglou[1], Lama AlAbdi[2], Muhammad Tehseen[1], Yossef Lopez de los Santos[1], Mansour Aldehaiman[1], Gloria Lopez-Madrigal[1], Norah Altuwaijri[2], Viviana Hermosilla Aguayo[3], Anna Pleyers[4], Maya Ayach[5], Ashraf Al-Amoudi[5], Jing Kai[1], Vlad-Stefan Raducanu[1], Bader Alhaddad[6], Edda Hofstätter[7], Amani Al-Amodi[1], Mais O. Hashem[2], Hessa S. Alsaif[8], Claudius Fazelnia[9], Firdous Abdulwahab[2], Amal Jaafar[2], Tarfa Alshidi[2], Rana Helaby[2], Adriana M. Montaño[10], Kara C. Klemp[11], Ellen Totten[11], Wesam Kurdi[12], Saly Eltohamy[13], Hadeel Abdollah E. Alrukban[14], Bashayer Alawam[15], Licia Selleri[3], Davor Lessel[16,17], Dimitrios Kapogiannis[18,19], Samir M. Hamdan[1], Stephen R. Braddock[11], Fowzan S. Alkuraya[1,2,6,20*], Jasmeen S. Merzaban[1,21*]

[1]Bioscience Program, Biomedical Sciences Division, King Abdullah University of Science and Technology (KAUST), Thuwal, Saudi Arabia

[2]Department of Translational Genomics, Genomic Medicine Centre of Excellence, King Faisal Specialist Hospital and Research Center, Riyadh, Saudi Arabia

[3]Program in Craniofacial Biology, Department of Orofacial Sciences, Department of Anatomy, Institute for Human Genetics, Eli and Edythe Broad Center of Regeneration Medicine and Stem Cell Research, University of California, San Francisco, San Francisco, CA, USA

[4]Institute of Human Genetics, University Hospital Salzburg, Paracelsus Medical University, 5020 Salzburg, Austria.

[5]KAUST Core Labs, KAUST, Thuwal, 23955-6900, Kingdom of Saudi Arabia

[6]Lifera Omics, Riyadh, Saudi Arabia

[7]Division of Neonatology, Department of Pediatrics and Adolescent Medicine, Paracelsus Medical University, Salzburg 5020, Austria

[8]Wellness and Preventative Medicine Institute, Health Sector, King Abdulaziz City for Science and Technology (KACST), Riyadh 11442, Saudi Arabia

[9]Department of Obstetrics and Gynecology, Paracelsus Medical University, 5020 Salzburg, Austria

[10]Department of Pediatrics, and Biochemistry and Molecular Biology, School of Medicine, Saint Louis University, St. Louis, MO, USA

[11]Department of Pediatrics Saint Louis University School of Medicine, St Louis, MO, USA

[12]Department of Obstetrics and Gynecology, King Faisal Specialist Hospital and Research Center, Riyadh, Saudi Arabia

[13]Dr. Sulaiman Al Habib Medical Group, Riyadh, Saudi Arabia

[14]Department of Pediatrics, Prince Sultan Military Medical City, Riyadh, Saudi Arabia

[15]Department of Pediatrics, King Fahad Hospital of the University, Al-Khobar, Saudi Arabia

[16]Institute of Human Genetics, University of Regensburg, 93053 Regensburg, Germany

[17]Institute of Clinical Human Genetics, University Hospital Regensburg, 93053 Regensburg, Germany

[18]Laboratory of Clinical Investigation, Intramural Research Program, National Institute on Aging, National Institutes of Health (NIA/NIH), Baltimore, Maryland, USA

[19]Department of Neurology, Johns Hopkins School of Medicine, Baltimore, Maryland, USA

[20]College of Medicine, Alfaisal University, Riyadh, Saudi Arabia

[21]KAUST Center of Excellence for Smart Health (KCSH), King Abdullah University of Science and Technology (KAUST), Thuwal, Saudi Arabia

*Correspondence: falkuraya@liferaomics.com.sa & jasmeen.merzaban@kaust.edu.sa

Antibody	Vendor	Catalog number
Anti-Vps25	Santa Cruz	sc-271648
Anti-Vps36	Thermo Fisher	PA5-60561
Anti-Vps22	Novus Biologicals	NBP2-15125
Anti-SQSTM1	Thermo Fisher	GT1478
Anti-CD81	Abcam	Ab79559
Anti-CD63	Thermo Fisher	10628D
Anti-DNA polymerase gamma	Cell Signaling	13609
Anti-Lamin A/C	Cell Signaling	4777
Anti-mouse IgG, HRP	Cell Signaling	7076
Anti-rabbit IgG, HRP	Cell Signaling	7074

Table S1: Antibodies used for the western blot analysis.

Supplementary Figures

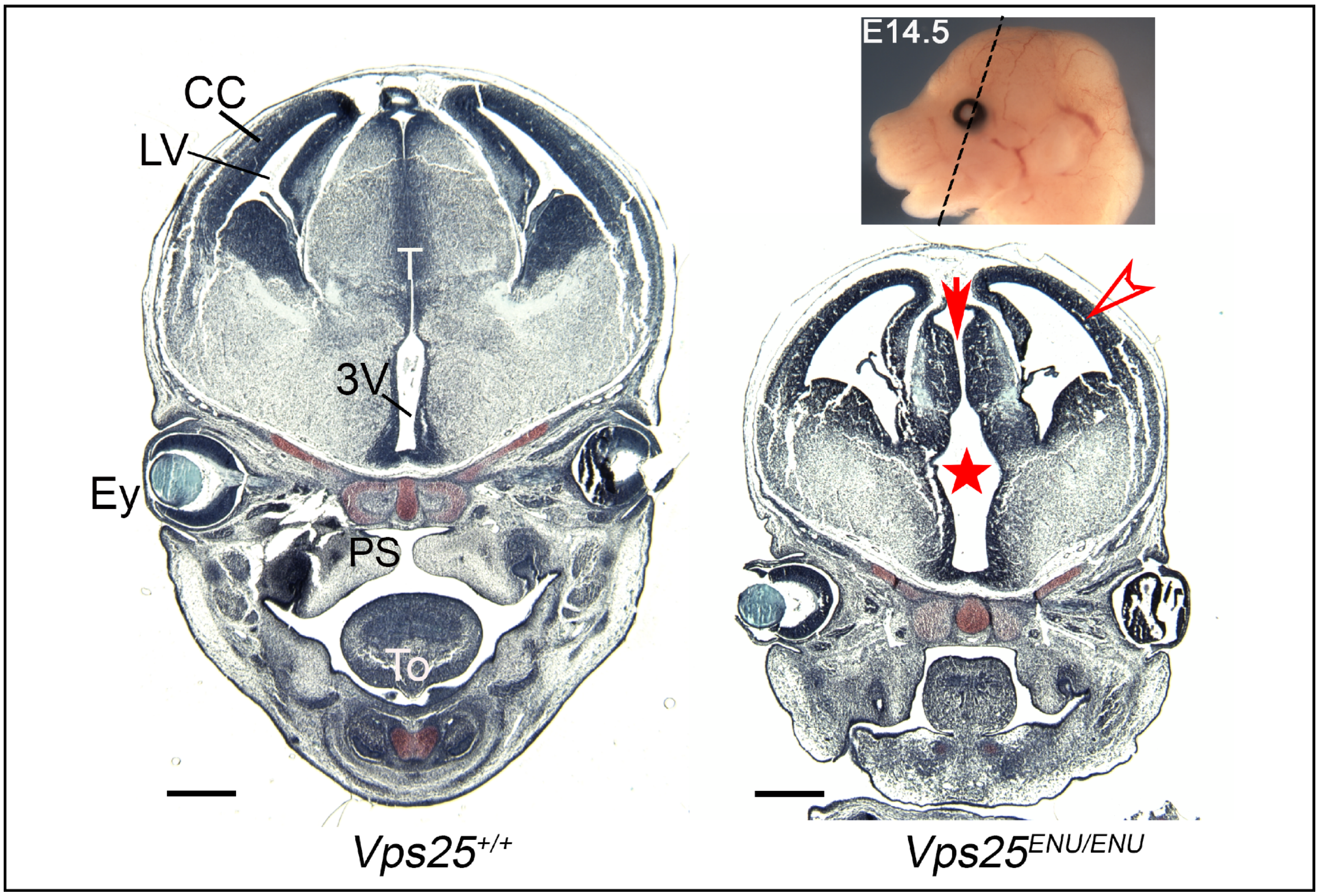

Fig. S1: Masson's Trichrome-stained coronal sections of heads from E14.5 Vps25 constitutive hypomorphic (*$Vps25^{ENU/ENU}$*; **right panel**) and wildtype control (*$Vps25^{+/+}$*; **left panel**) mouse embryos. The *$Vps25^{ENU/ENU}$* mutant embryo shows strikingly dilated lateral ventricles with thinned cerebral cortex (empty red arrowhead) as compared to wildtype control. In addition, the *$Vps25^{ENU/ENU}$* thalamus is markedly hypoplastic (red arrow) with an enlarged third ventricle (red star). Plane of the sections across the eye shown as a dashed black line in the representative mouse embryonic head (**top right panel**). Cerebral Cortex, CC; Eye, Ey; LV, Lateral Ventricle; PS, Palatal Shelf; T, Thalamus; To, Tongue; 3rd Ventricle,3V. Scale bar, 500 µm.

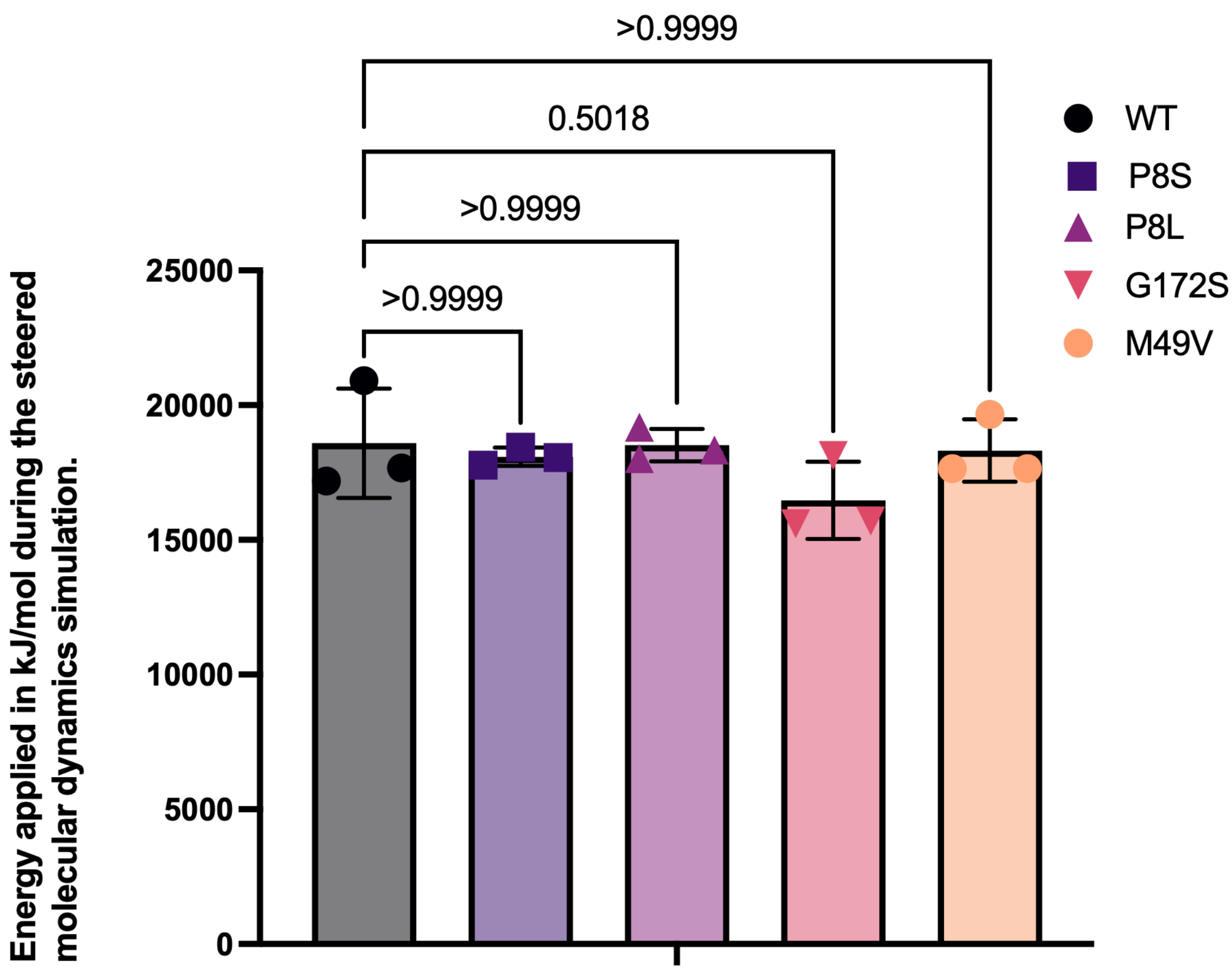

Fig. S2: Steered Molecular Dynamics simulation for the WT ESCRT-II complex and the P8S, P8L, M49V, and G172S mutants. This analysis reports on the energy required to separate the VPS25 protein from the protein interface with VPS22/VPS36. Error bars represent the standard deviation of a triplicate. A one-way ANOVA was used to measure the statistical difference of these results, applying a Bartlett correction. No significant differences among the protein variants were observed.

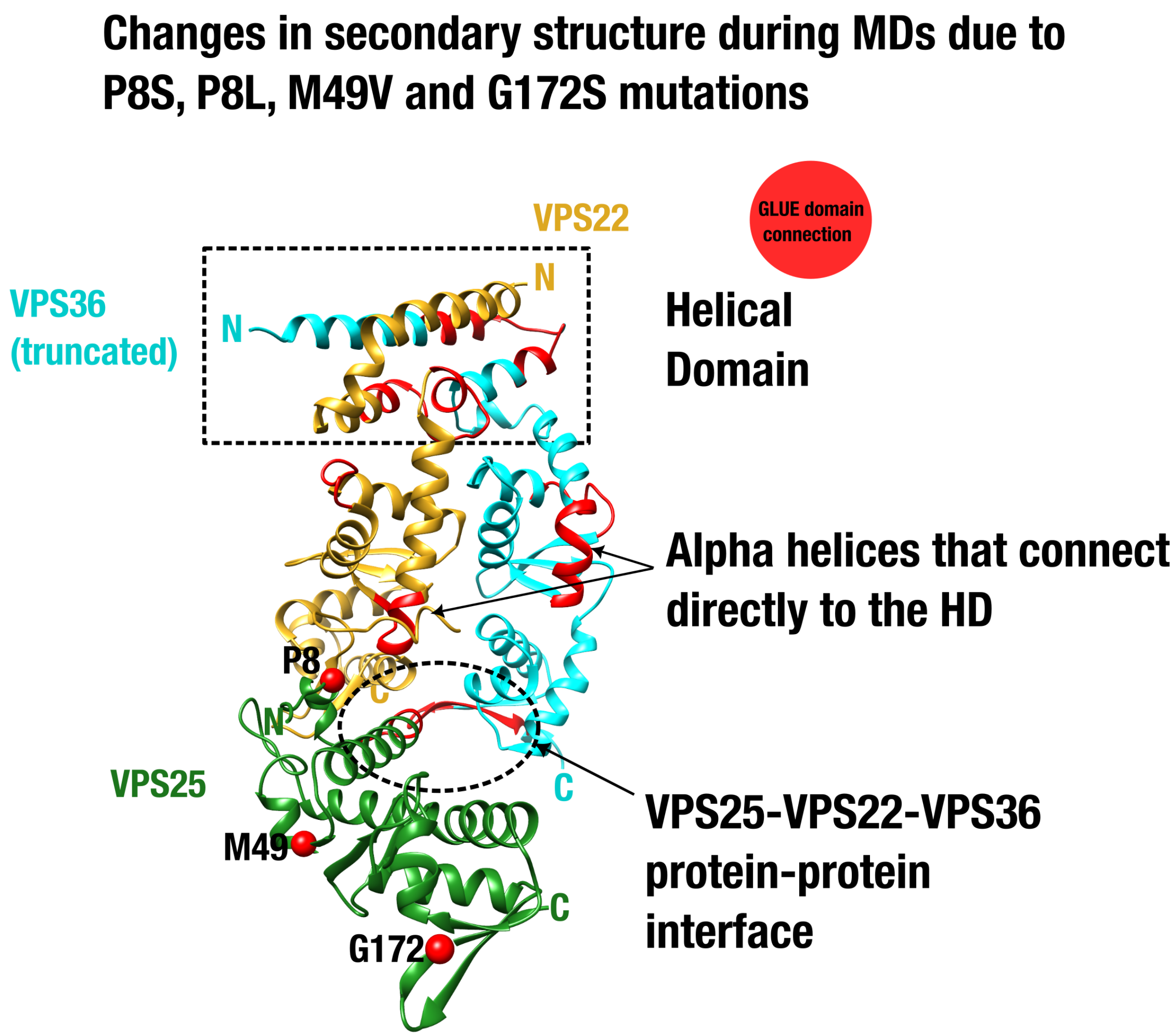

Fig. S3: Changes in the secondary structures of the three protein members of the ESCRT-II complex may be linked to the decreased HD molecular dynamics observed in the VPS25 P8S, P8L, M49V, and G172S mutants. Perturbations were found in the HD, alpha helix connecting VPS36 to the ESCRT-II core, and the VPS25-VPS36-VPS22 protein interface. Mutation positions 8, 49, and 172 are marked with red spheres. The changes observed in the secondary structure during the MD simulations are highlighted in red.

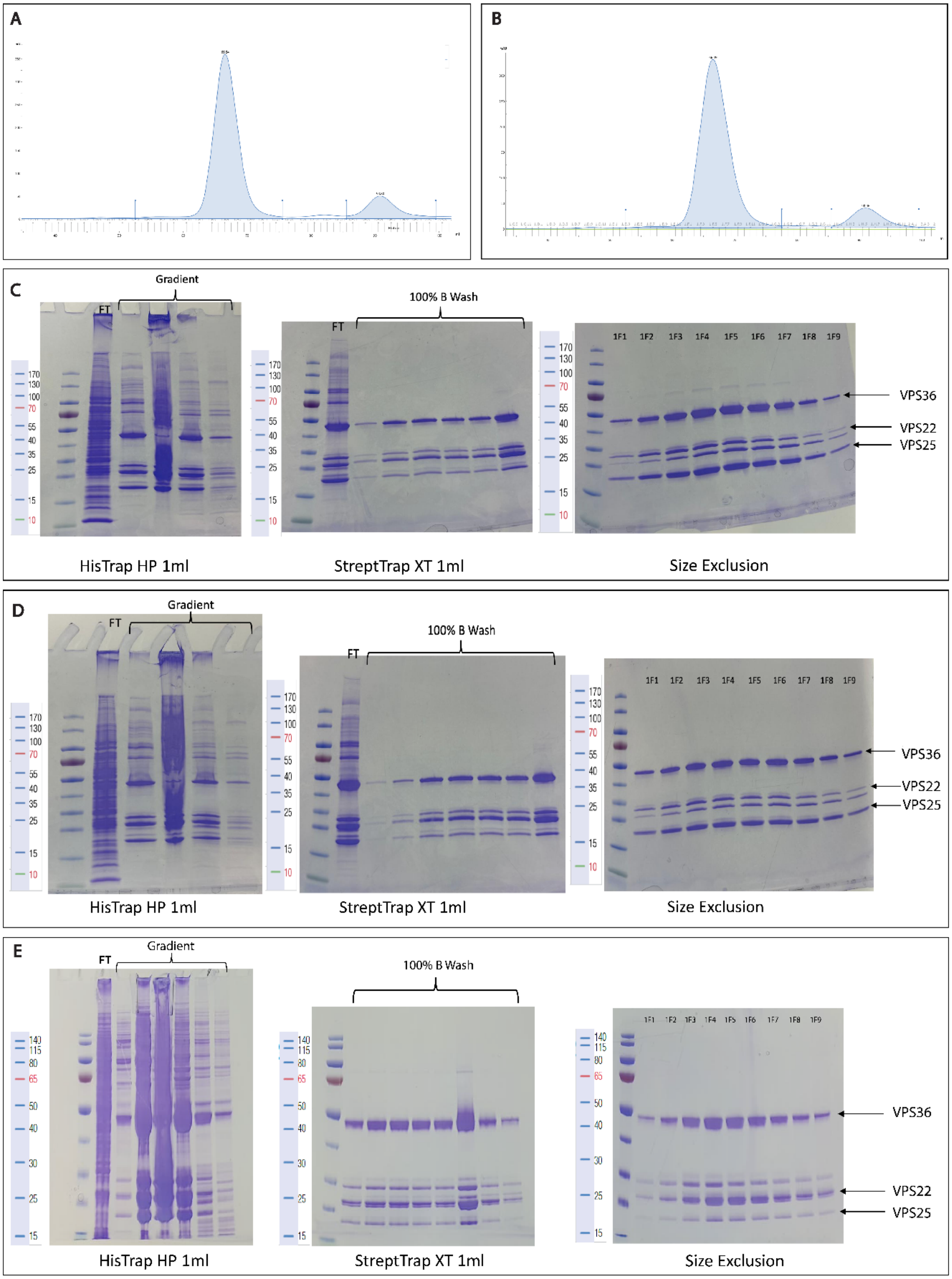

Fig. S4: Purification of human recombinant ESCRT II VPS25^{WT} and ESCRT II VPS^{P8S} mutant complex from Sf9 insect cells. **(A-B)** Representative chromatograms from size exclusion chromatography from ESCRT II Vps25^{WT} **(A)** and from ESCRT II VPS25^{P8S} mutant **(B)**. **(C)** 4-20% SDS-PAGE gels showing the different steps

of purification of ESCRT II VPS25^{WT} complex from HisTrap HP, StrepTrap XT and size exclusion columns. **(D)** 4-20% SDS-PAGE gels showing the different steps of purification of ESCRT II VPS25^{P8S} mutant complex from HisTrap HP, StrepTrap XT and size exclusion columns. **(E)** 4-20% SDS-PAGE gels showing the different steps of purification of ESCRT II VPS25^{M49V} mutant complex from HisTrap HP, StrepTrap XT and size exclusion columns.

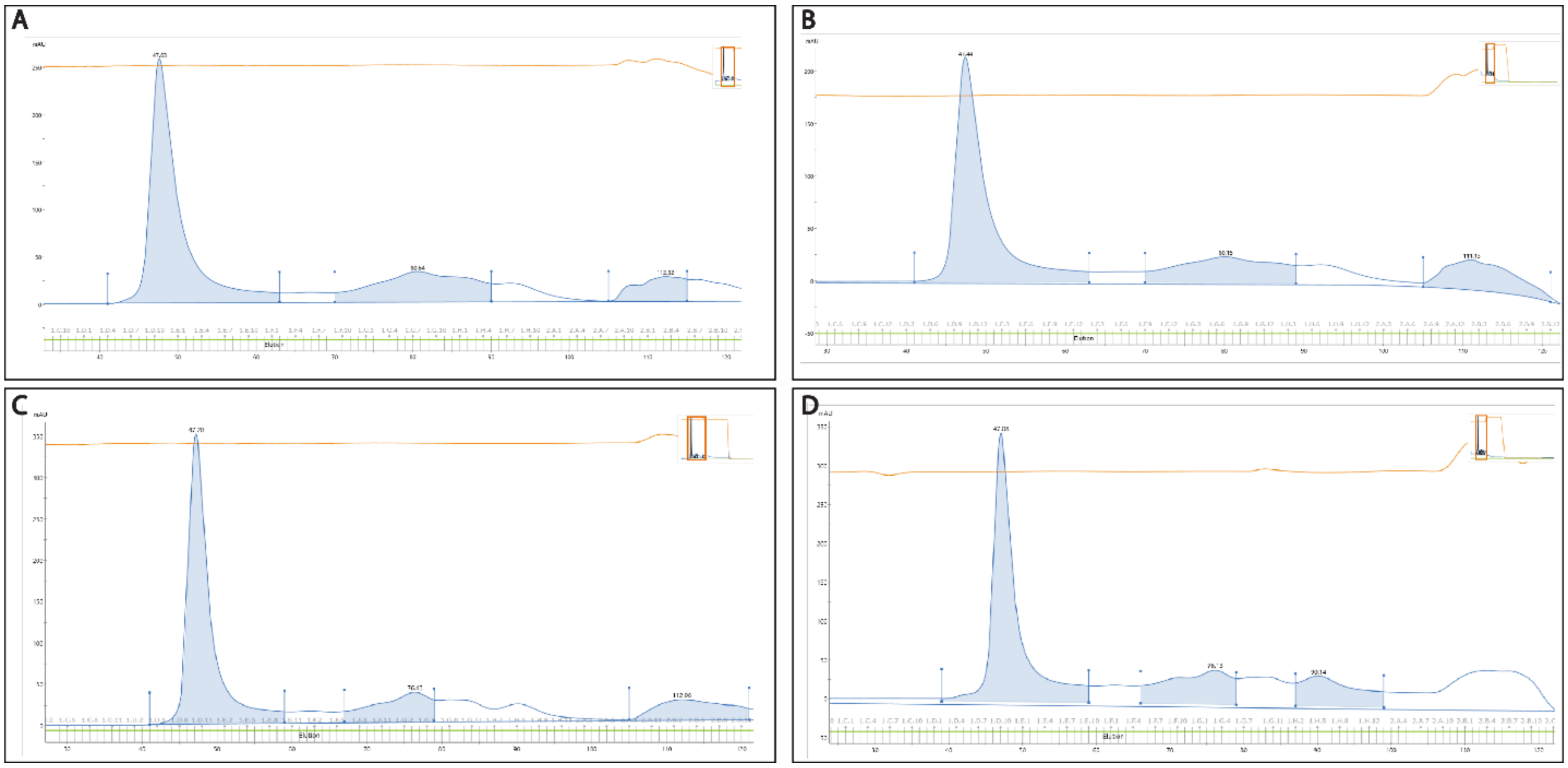

Fig. S5: Size exclusion chromatography results from VPS25^{WT1} (**A**), VPS25^{WT2} (**B**), VPS25$^{P8S/P8S}$ (**C**) and VPS25$^{P8L/G172S}$ (**D**) patient-derived cells.

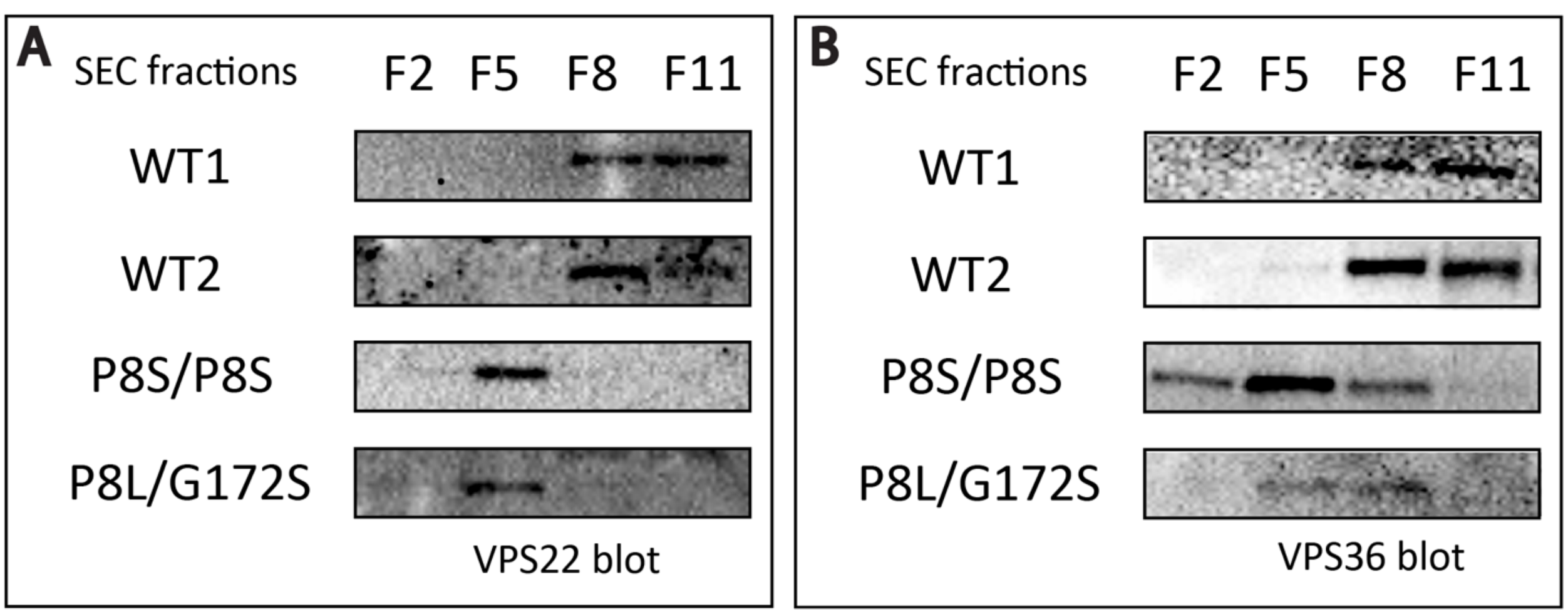

Fig. S6: Western blots of the fractions F2, F5, F8 and F11 produced by the size exclusion chromatography of total lysate from each different patient. (**A)** Blot for VPS22. (**B)** Blot for VPS36.

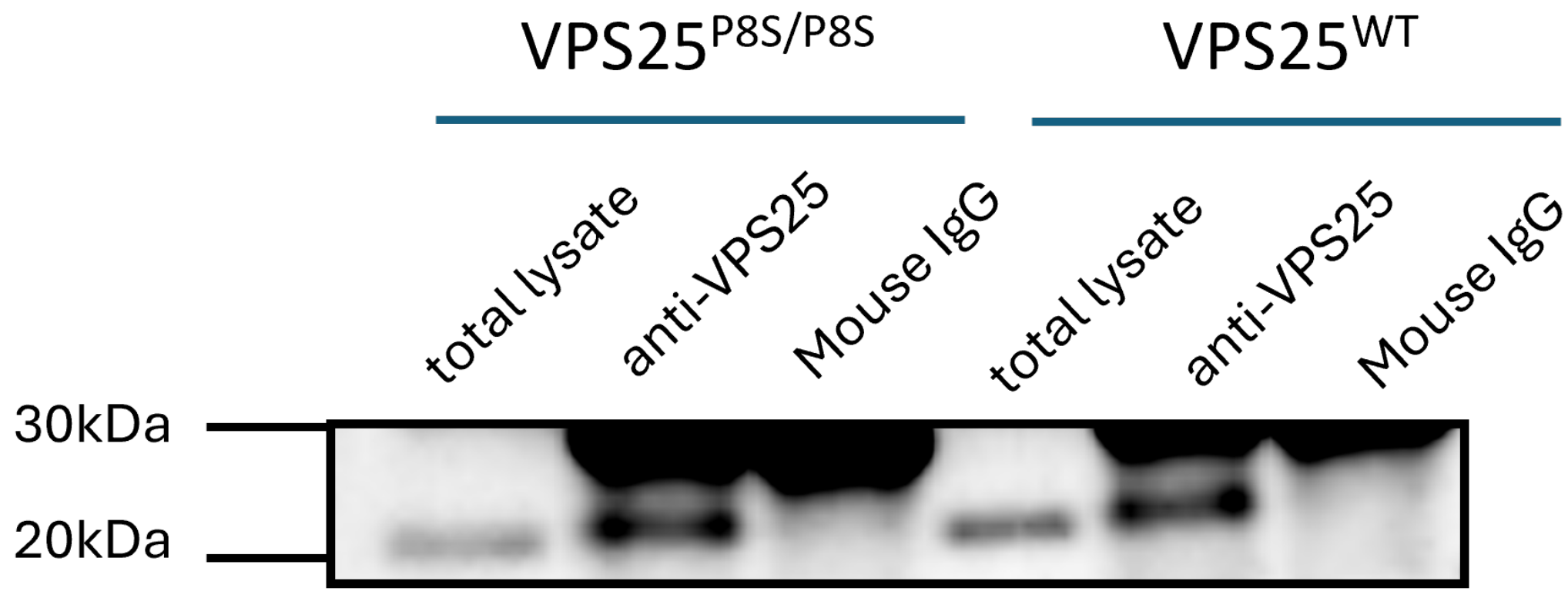

Fig. S7: Western blot analysis of VPS25 co-immunoprecipitation. Total lysate corresponds to 10 % input of the total cell lysate. Anti-VPS25 corresponds to 10% input of the immunoprecipitation product using the anti-VPS25 (D-6, Santa Cruz). Mouse IgG corresponds to 10% input of the immunoprecipitation product using the mouse IgG antibody (ab188776, Abcam). Samples from control patient cells (right panel) and VPS25-mutated patient cells (left panel) were analyzed; successful pull-down of VPS25 was confirmed in both. The intense bands on the top part of the membrane belong to the light chain of the antibodies used for immunoprecipitation.

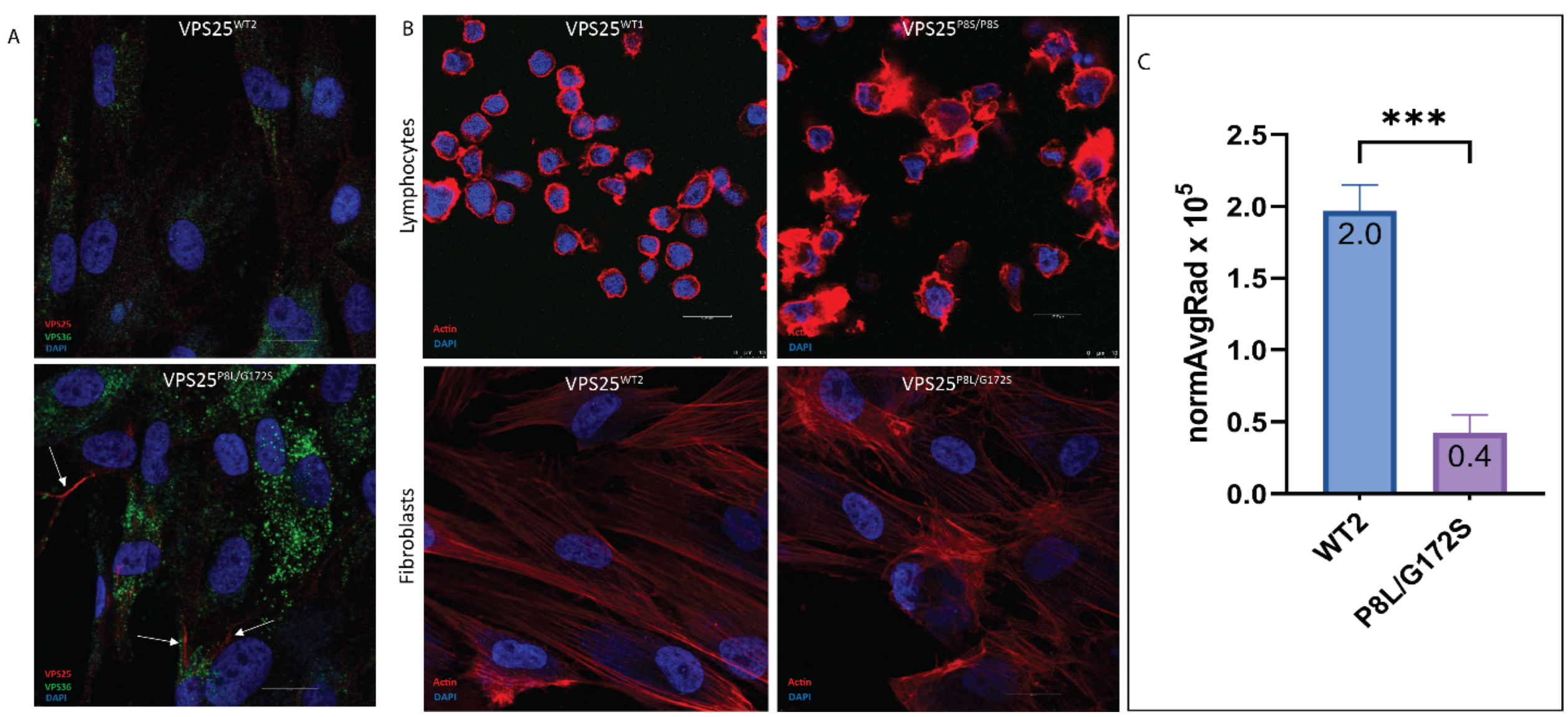

Fig. S8: Microscopy analysis of *VPS25*$^{P8S/P8S}$ and *VPS25*$^{P8L/G172S}$ patient-derived cells showed abnormal cytoskeleton. **(A)** Fibroblast cells derived from patients with (*VPS25*$^{P8L/G172S}$) or without VPS25 (*VPS25*^{WT}) mutations were stained for VPS25 protein using AF647-Vps25, VPS36 protein using AF488-Vps36 as well as DAPI. A higher concentration of VPS25 is observed on the edge of the cells in the VPS25$^{P8L/G172S}$ cells compared to wild-type. White arrows show dense clustering of VPS25 on the membrane proximal region of the *VPS25*$^{P8L/G172S}$ cells. (**B)** Fibroblast or Lymphocyte cells either from normal patients or patients with a mutant VPS25 protein variant were imaged using Leica SP8 confocal microscopy. Cells were stained with Alexa Fluor™ 647 Phalloidin for actin and, DAPI, dilactate for cellular DNA. Mutant cells show a more

disrupted and more irregular shape for actin filaments. (**C)** The degree of "parallelism" of the actin fibers in fibroblast cells was measured using an ImageJ plugin and represented as "normAvgRad".

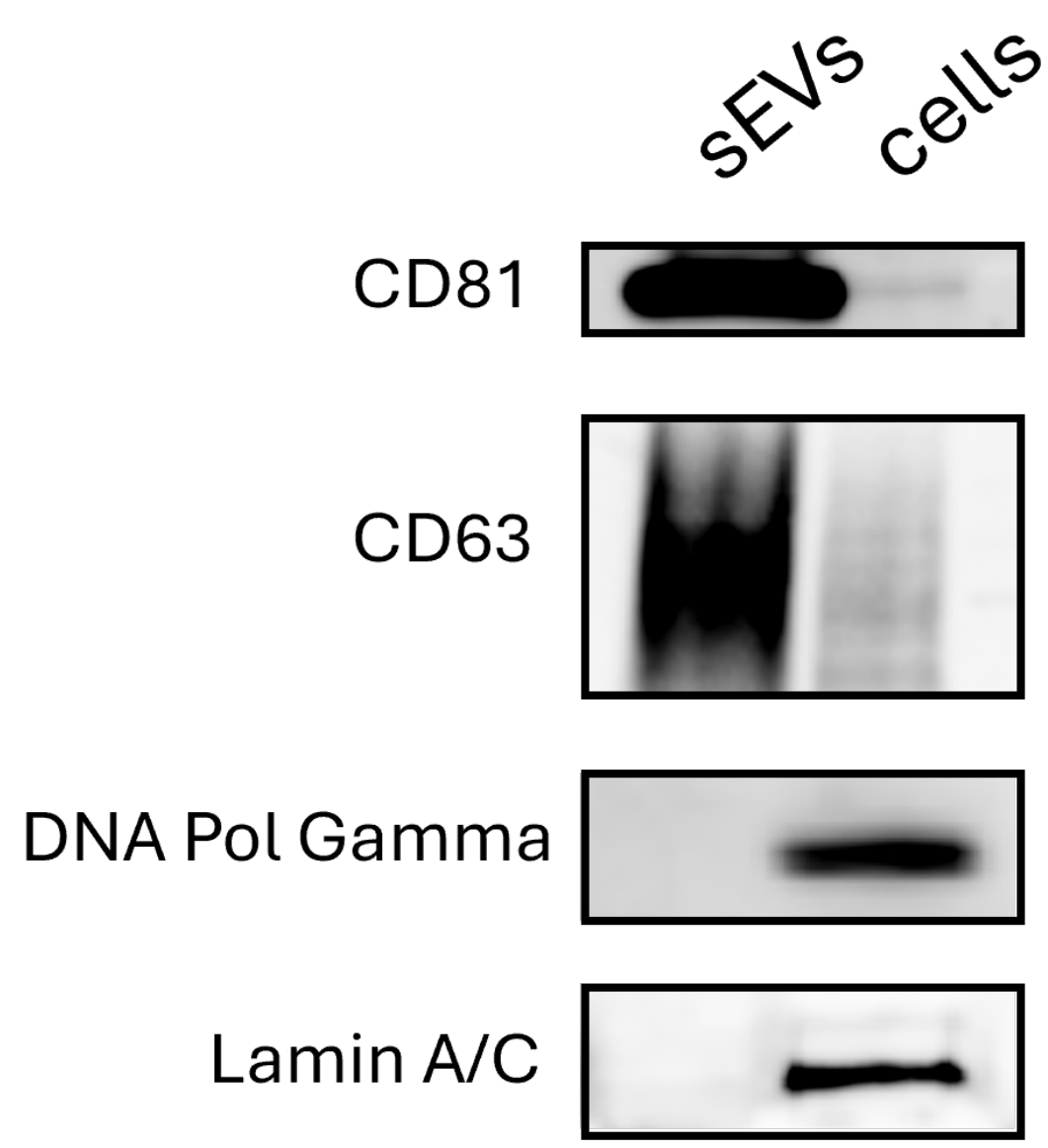

Fig. S9: Western blot analysis of protein enrichment in sEVs compared to parental cell lysates. Equal protein amounts were loaded to allow for direct comparison of marker abundance. CD81 and CD63 were utilized as positive markers to demonstrate sEV enrichment, while DNA Polymerase Gamma (mitochondria) and Lamin A/C (nucleus) were used as negative markers to confirm the absence of significant cellular contaminant carry-over in the sEV isolations

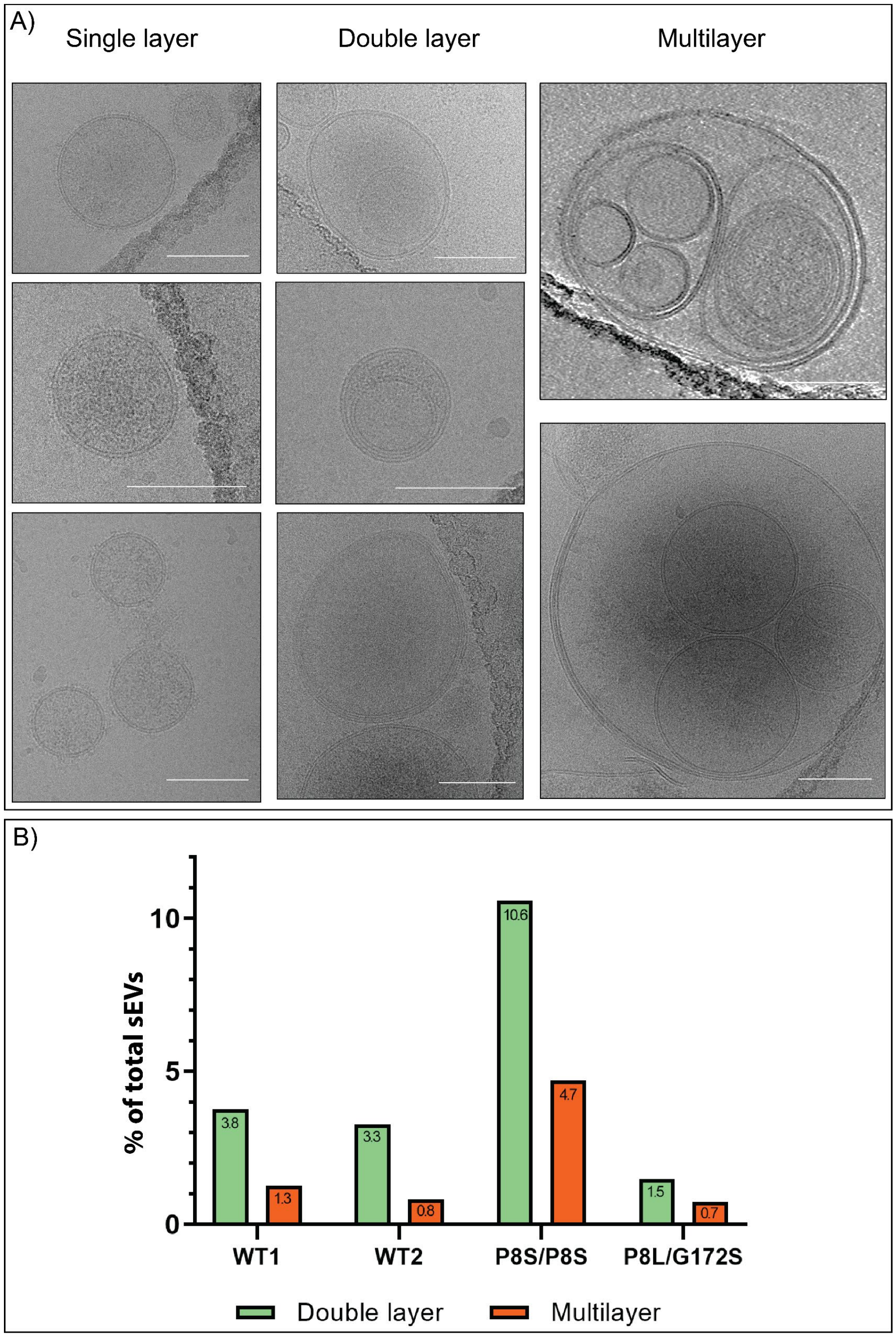

Fig. S10: Different patterns of morphology of small extracellular vesicles (sEVs) observed under Cryo-EM. **(A)** Different types of sEVs were observed, having one (single layer), two (double layer) or more (multiple layer) membrane layers. White scale bar indicates 100nm. **(B)** The percentages of sEVs displaying double layer membrane (green) or a multiple-layer membrane (orange) were different in the sEVs derived from wild-type lymphoblasts, compared to the ones derived from the *VPS25* mutant cells.

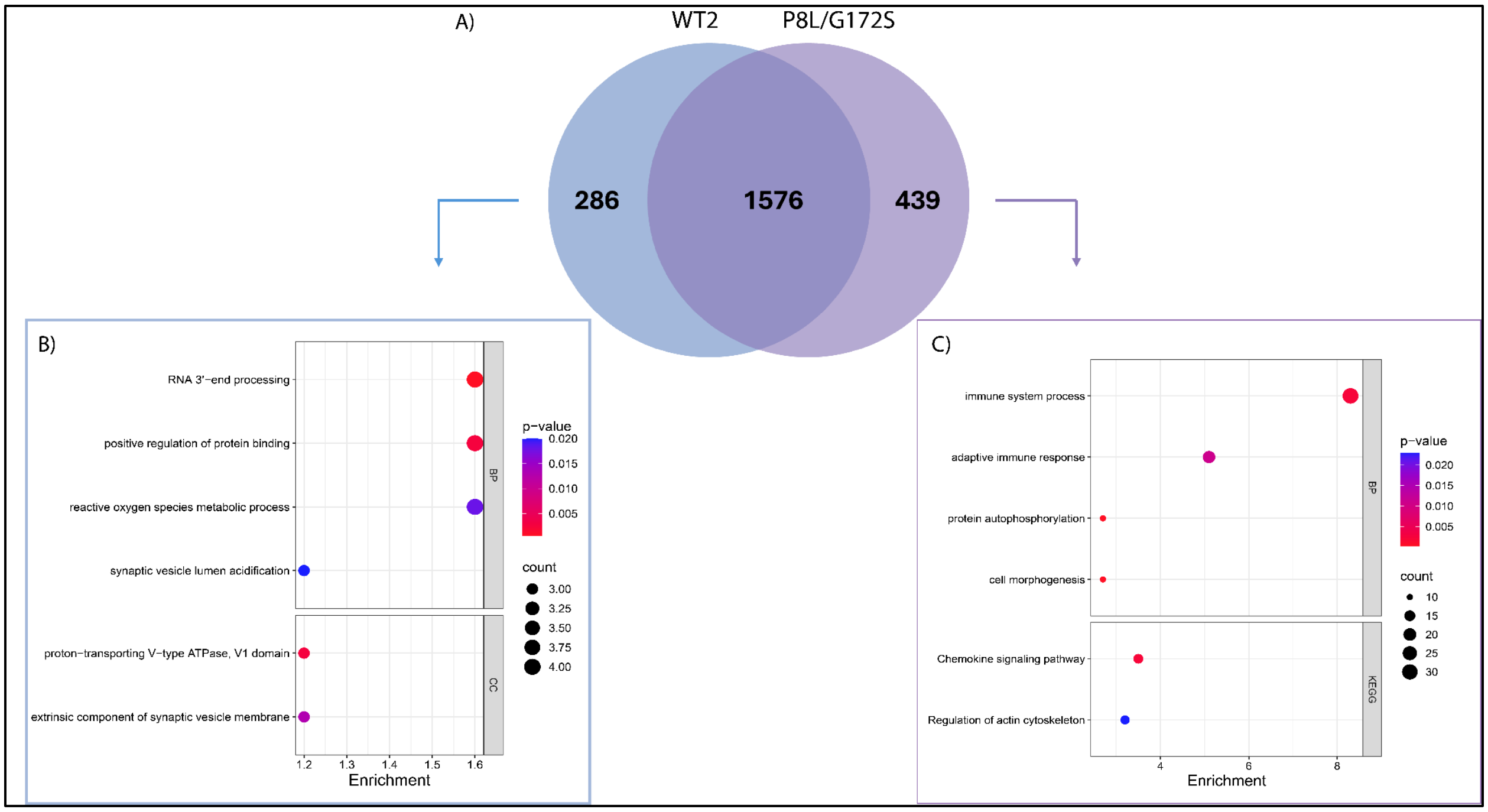

Fig. S11: (A) Protein cargo identified into sEVs derived from wild type (*VPS25*^{WT}) and mutated (*VPS25*$^{P8L/G172S}$) lymphoblasts. **(B)** Unique categories of functional analysis of the proteins identified only in the wild type derived sEVs. **(C)** Unique categories of functional analysis of the proteins identified only in the patient derived sEVs. BP: Biological Process, CC: Cellular Compartment, KEGG: Kyoto Encyclopedia of Genes and Genomes. The comparison of sEVs derived from *VPS25*$^{P8L/G172S}$ cells to sEVs derived from cells from WT2 patient is to align with the age and gender of the patient.
