# Supplementary material for: Recessive variants in ESCRT-II component VPS25 alter vesicular transport and small extracellular vesicle processing in lethal neonatal encephalopathy
Source: Genome Med. 2026 Sep 14;18:165. doi: 10.1186/s13073-026-01693-z (PMC13712088; doi:10.1186/s13073-026-01693-z)
Supplement: Supplementary file 2 [file 13073_2026_1693_MOESM2_ESM.pdf]

Additional file 2- Supplementary Clinical Data

Family 1 Patient 09DG01431 (IV:12) was born to first-cousin parents. The mother (III:3) previously lost five infants with a similar clinical presentation (see below): two from her first marriage and three from her current marriage. In total, six infants were lost across these two consanguineous unions with two brothers (III:6 and III:7). Ultrasound imaging of 09DG01431 at gestational age 21 weeks and 6 days revealed abnormally shaped cerebellum, normal cisterna magna, hypoplastic vermis, partial agenesis of the corpus callosum (CC), dilated cavum septum pellucidum, non-dilated lateral ventricles, head circumference (HC) 193.4 mm (30th centile), abdominal circumference (AC) 159.5 mm (20th centile), biparietal diameter (BPD) 51.4 mm, and femur length (FL) 39.1 mm (95th centile). The baby was born at 40 weeks of gestation via normal vaginal delivery. She was admitted to NICU with severe hypotonia and died at the age of 4 months due to cardiac arrest.

We were able to obtain the medical records for two siblings who died of similar presentations: brother of 09DG01431 was diagnosed with seizure disorder and absence of corpus callosum. The pregnancy history was normal. Delivery was at 40 weeks of gestation via normal vaginal birth. His Apgar score was 4, 7, and 8 at 1, 5, and 10min, respectively and he was admitted to the NICU due to severe axial hypotonia and suspected hypoxic ischemic encephalopathy. His NICU course was notable for apnea, bradycardia and seizures. The patient was dysmorphic with micrognathia. He was also microcephalic and spastic in both upper and lower limbs. Brain imaging using MRI revealed the absence of the corpus callosum. Electroencephalogram revealed cortical dysfunction which was diffuse, involving both hemispheres, and cortical irritability, involving multiple regions with both hemispheres. Tandem MS was unremarkable. He remained profoundly delayed and ventilator-dependent until he arrested at age 8 months and died. The pregnancy of the affected sister of 09DG01431 was uncomplicated. She was born at term with normal Apgar scores. Her birth weight 2.75 kg, length 49 cm, and head circumference 33 cm were within normal limits, and she had no dysmorphic features. However, her neonatal exam was notable for poor feeding, hypotonia, and hyporeflexia. Thus, she was transferred to NICU for nasogastric tube feeding and further evaluation, which revealed brain atrophy on brain CT. Her course in the NICU was notable for poor sucking and hypotonia. Ultimately, she died at age 4 months due to prolonged apnea and cardiac arrest.

Patient 19DG2560 (IV:2, maternal cousin of 09DG01431), was a product of third-cousin parents with a history of two previous neonatal deaths similar to what has been observed in the above-described cousins, in addition to one sibling with ADHD. Delivery was at 36 weeks of gestation via normal vaginal birth. At delivery, the baby was limp, cyanosed with poor respiratory effort (APGAR score was 4, 7, and 8 at 1, 5, and 10min, respectively). He was resuscitated and admitted to NICU where he developed convulsions at 14hrs of

life and these were initially controlled with phenobarbitone. However, on day three, he developed convulsions and right lung collapse. Full sepsis workup and ECG were unremarkable. Brain imaging by u/s showed dilated lateral ventricles more so on the right side. He was frequently admitted to NICU and we have limited data on the developmental millstones. He had recurrent generalized seizures with eyes rolling. At 4 months of age, he had an episode of cyanosis and bradycardia with bradypnea, arrested and died. We were able to obtain medical and MRI reports for deceased brother of 19DG2560 (F1:IV:1). He was delivered vaginally at full term, APGAR score was 9 and 9 at 1 and 5 minutes. At 12 hrs of age, he developed an episode of apnea and desaturation the first of many to follow necessitating multiple admissions. He later developed seizures in the form of staring, tonic posturing of both upper limbs with pupillary dilations. He was treated with Keppra and phenobarbital.

Family 2 The proband (F2:III:3) was born at 37 weeks, 2 days to a 34-year-old G3P3 mother via C-section due to breech presentation. Exposures during pregnancy included prenatal vitamins with iron, acetaminophen, diphenhydramine, magnesium, ranitidine, and Rhogam. She denied tobacco, alcohol, or other substance use. Maternal blood type was A-. Pregnancy was complicated by prenatal diagnosis of ventriculomegaly with suspected aqueductal stenosis, polyhydramnios, decreased fetal movement, and a maternal UTI. Amniocentesis was sent for CMA as well as CMV and toxoplasmosis, which were all negative. Ultrasound at 20 weeks was normal, but by week 34, the ultrasound revealed evidence of hydrocephalus. She was born via C-section due to breech presentation. Birth weight 3380g (84%), length 47 cm (36%), and head circumference 34 cm (72%). Apgar scores were 6 and 8 at one and five minutes, respectively. There was a 3-vessel umbilical cord. Physical exam revealed a hypotonic infant with low set ears who was otherwise non-dysmorphic. At the time of delivery, she was noted to have abnormal movements consistent with seizure activity and she was transferred to the NICU. EEG and MRI were ordered. EEG was indicative of severe diffuse cerebral dysfunction, with subclinical epileptic foci in the right occipital region and areas of subcortical myoclonus. The family history was noteworthy for a maternal first cousin-once removed diagnosed with hydrocephalus at age 21, requiring surgery. No other family history of birth defects, developmental delay, intellectual disability, congenital blindness/deafness, recurrent pregnancy loss or consanguinity. Initial hospital course noted stable hydrocephalus with cerebral volume loss and no evidence of increased intracranial pressure. Seizures were controlled with anticonvulsants. Due to respiratory instability, she underwent tracheostomy at 6 weeks of age. Gastrostomy and Nissen fundoplication were also performed. Echocardiogram was normal. She was discharged home at 3 months of age.

At 4 months of age, head circumference was rapidly increasing and a ventriculoperitoneal shunt was placed. Subsequent medical issues include chronic respiratory failure with ventilator dependence, cortical visual impairment with optic nerve hypoplasia, moderate

to severe sensorineural hearing loss, SIADH, spastic quadriplegic cerebral palsy, persistent truncal hypotonia, and global developmental delay. She developed recurrent bullous lesions on her skin of uncertain etiology. At 3 years 7 months, weight was 17 kg (78%) and length 81.3 cm (<1%).

Family 3: The patient´s history revealed no relevant information, the 20 weeks scan showed no fetal malformation. The fetal ultrasound performed at 34+5 gestational weeks showed severe bilateral ventriculomegaly (23mm left, 23mm right) and a dilated third ventricle (6,1mm) with normal cephalic circumference. The cavum septi pellucidi was visible, the corpus callosum could not be visualized due to the fetal position. Fetal Doppler of the umbilical and the cerebral artery including peak systolic velocity showed no signs of fetal compromise or fetal anemia. Cytomegaly virus and toxoplasmosis PCR, also tested in the amniotic fluid, showed negative results. Maternal serological testing for relevant infections (TORCH) provided also no pathological findings. Fetal magnet resonance imaging (MRI), indicated, beside the known ventriculomegaly, a subtotal agenesis of the corpus callosum with colpocephaly and occipital rarefaction of the white matter. No signs for intracranial hemorrhage or tumorous lesions could be detected.

The pregnancy was monitored weekly and at 37+4 weeks, ultrasound showed increasing size of both lateral ventricles (27mm) and severe polyhydramnios. Due to assumed aggravation of the fetal situation and the remote residence of the patient, induction of labor was started at 37+6 weeks. On the second day of uneventful induction, the patient had sudden premature rupture of the membranes (PROM) with acute bloodily amniotic fluid and a sudden onset of labor. The patient was immediately transferred to the delivery room and delivered 20 minutes after PROM a severely compromised asphyctic female newborn. APGAR scores 0/4/5 and pH 6,97

The newborn was found pale, atonic and asystolic. Immediate, chest compressions were started. The newborn was intubated and an intraosseous access was applied. L-adrenaline and volume bolus were given. After 5 minutes, sufficient cardiocirculation was obtained; but the infant showed no own breathing. Emergency erythrocyte transfusion was performed. The initial umbilical arterial blood work showed: pH 6,97; CO2 79,7 mmHg, Gluc. 64 mg/dl, Lac 10,1 mmol/l, HCO 18,3 mmol/l, BE -14,0 mmol/l, Hb 11,1 g/dl.

The infant was transferred to the NICU. Hypothermic treatment (rectal temperature of 34.0 – 34.5 °C) was started immediately as the premature abruption of the placenta had lead to a severe asphyxia with myocardial insufficiency and severe coagulation failure with lung bleeding. Catecholamine treatment including epinephrine, arterenol and dobutamine was administered. The hypothermic treatment had to be interrupted because of a cardiogenic shock for a few hours. After stabilisation hypothermia was continued. After birth the infant showed anuria therefore peritoneal dialysis was successfully established on the second day of life, A urine excreation by the infant could never be

achieved despite maximal diuretic treatment (Furosemide, Spironolactone, Etacryn Acid). After a total of 72 hours hypothermia was terminated. Despite no analgesic medication the infant never showed actual signs of own breathing and only very little spontaneous movements. The aEEG showed a burst-suppression pattern without any sedating drugs. Protective reflexes were missing. The MRI showed a picture of a severe hypoxic cerebral damage with symmetrical ischemic diffusion restrictions in both thalami and the central regions on both sides. Furthermore there was the ventriculomegaly in both rear horns with intraventricular hemorrhage especially on the right. The cerebral parenchym was markedly diminished in the parieto occipital region. As on the prenatal image there was an agenesis of the corpus callosum.

Family 4: The proband is a male boy who was a product of normal vaginal delivery at 36 weeks of gestation, APGAR score were 6 / 9 at 1 and 5 minutes respectively needed initial steps of resuscitation , IPPV for 30 seconds and naloxone given in delivery room , birth weight was 2.78 kg . Examination at delivery room was unremarkable apart from hypospadias with head circumference at birth 31 cm (10th centile). Parents are positive for consanguinity. During anti-natal follow up ultrasound was performed which showed ventricular dilatation. Brain ultrasound was done as there is history of bilateral ventriculomegally antenatally and reported as dilated both lateral ventricles, no IVH is seen and corpus callosum was absent.

The baby had very poor sucking and developed neonatal jaundice at age of 17 hours of life so admitted to neonatal intensive care unit. On 2nd day of life, baby developed focal convulsions in the right lower limb then the left side of the body, both events were self-aborted, and baby received a loading dose of Keppra as per pediatric neurologist. The proband received Midazolam before this event for sedation pre MRI procedure. EEG was done for 12 hours with normal result for this age. Brain MRI was reported as lateral ventricular parallelism with upturned frontal horns, dilated lateral ventricles posteriorly mainly along the trigone and occipital horn in keeping with posterior colpocephaly. Persistent eversion of cingulate gyrus. High-riding third ventricle reaching up to the interhemispheric fissure with these appearances are in keeping with agenesis of the corpus callosum also there is simplified gyral pattern noted in the study. The proband was also diagnosed with clinical mild hypotonia during admission and baby needed Nasogastric tube for feeding , which improved later on and baby was able to tolerate full oral feed upon discharge from neonatal intensive care unit.

On day 9 of life, the proband had convulsions so he was started on maintenance dose of keppra. He had soft heart murmur grade II; cardiac echo was reported as moderate Atrial Septal Defect Secundum, mild tricuspid regurgitation and moderate Patent Ductus Arteriosus. The G6PD level was sent due to neonatal jaundice and came low level G6PD: 1.1 (Deficient) and samples were sent for WES as per neurologist recommendation to rule out lissencephaly. WES reported a heterozygous variant in HK1 of uncertain

significance NM_001322365.2:c.1928A>G;p.(Gln643Arg). The proband currently resides in the NICU with minimal developmental millstones achieved (supported setting and standing. He has head control, mild rolling over, and he smiles but there is significant speech delay). He has frequent seizures.

His brother (IV:2) is four months old currently residing at the NICU due to feeding difficulties. He has mild head control and absence speech (only cooing). He smiles with mild rolling over and has not experienced seizures thus far. He was born full term, by NSVD and antenatal US at 22 weeks revealed absent corpus callosum. Initially, he was doing well until the age of 2 months when he developed a dry cough, shortness of breath, decreased activity, and decreased oral intake. He was then admitted to PICU due to fluid-responsive septic shock presumed due to hypoactivity and increased work of breathing. There was no history of fever (on prophylactic acetaminophen post-vaccination), seizures, cyanosis, choking, vomiting, or sick contacts. He had mild non-bloody diarrhea.

Family 5: The proband was a 2 years old male born with birth weight 3.2 kg, height 45cm, and head circumference of 35cm. He was found to have atrial septal defect (4.5mm) and ventricular septal defect (mid muscular 2mm) and patent ductus arteriosus. He has fine and gross motor delays as well as speech delay. Antenatal history was notable for fetal MRI which showed absent corpus callosum, cerebellum hypoplasia, ventriculomegaly. Brain MRI at two days of life showed complete agenesis of corpus callosum with colpocephaly and parallel appearance of the lateral ventricles. There is also a possibility of white matter injury/ PVL. There is also generalized cerebral atrophy. He was also spastic (hyperreflexia) and suffered from muscle weakness and seizures. He had hypoglycemia which was resolved and there are no notable dysmorphic features. Family history is notable of one similarly affected sister who passed away at the age of 9 months.
