# Supplementary material for: Recessive variants in ESCRT-II component VPS25 alter vesicular transport and small extracellular vesicle processing in lethal neonatal encephalopathy
Source: Genome Med. 2026 Sep 14;18:165. doi: 10.1186/s13073-026-01693-z (PMC13712088; doi:10.1186/s13073-026-01693-z)
Supplement: Supplementary file 3 [file 13073_2026_1693_MOESM3_ESM.pdf]

Additional file 3- Full-length blots

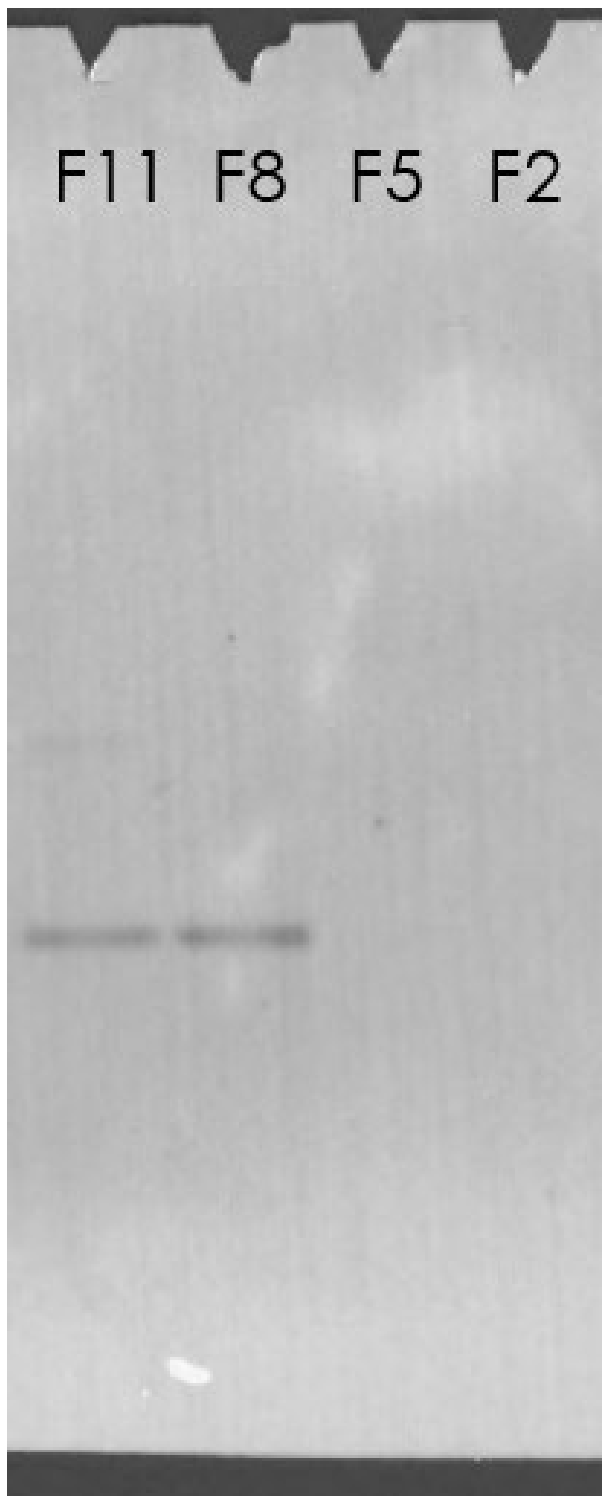

From Figure 3C, WT1

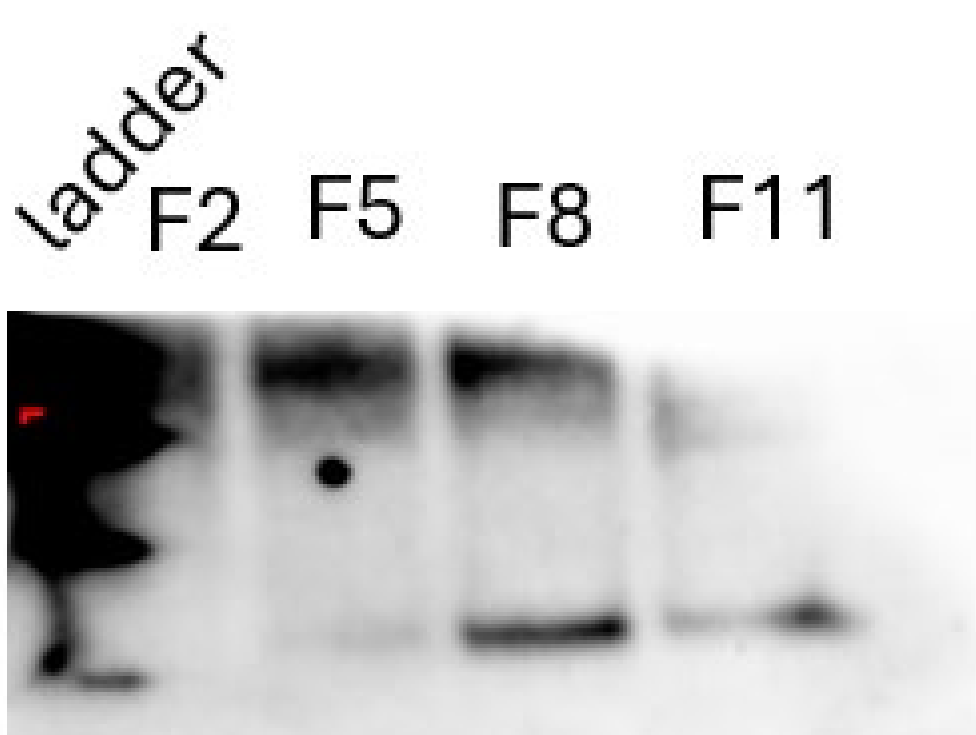

From Figure 3C, WT2

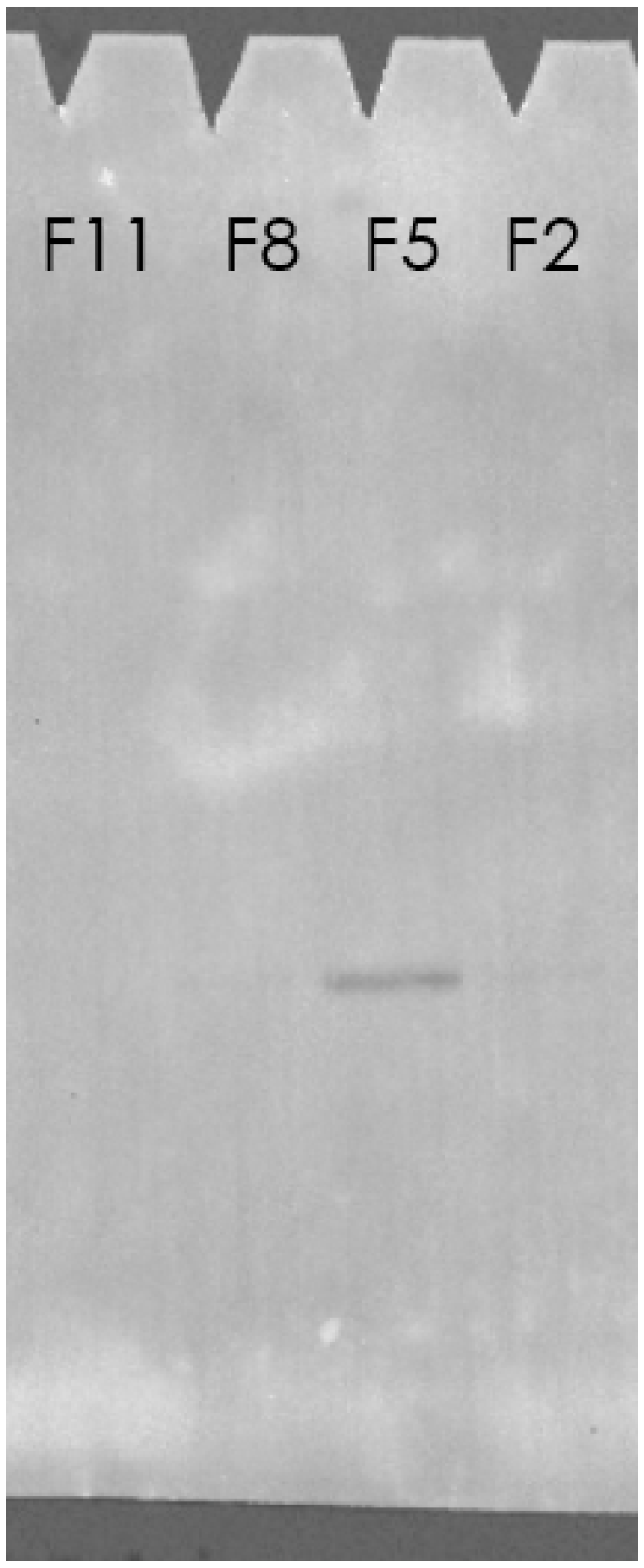

From Figure 3C, P8S/P8S

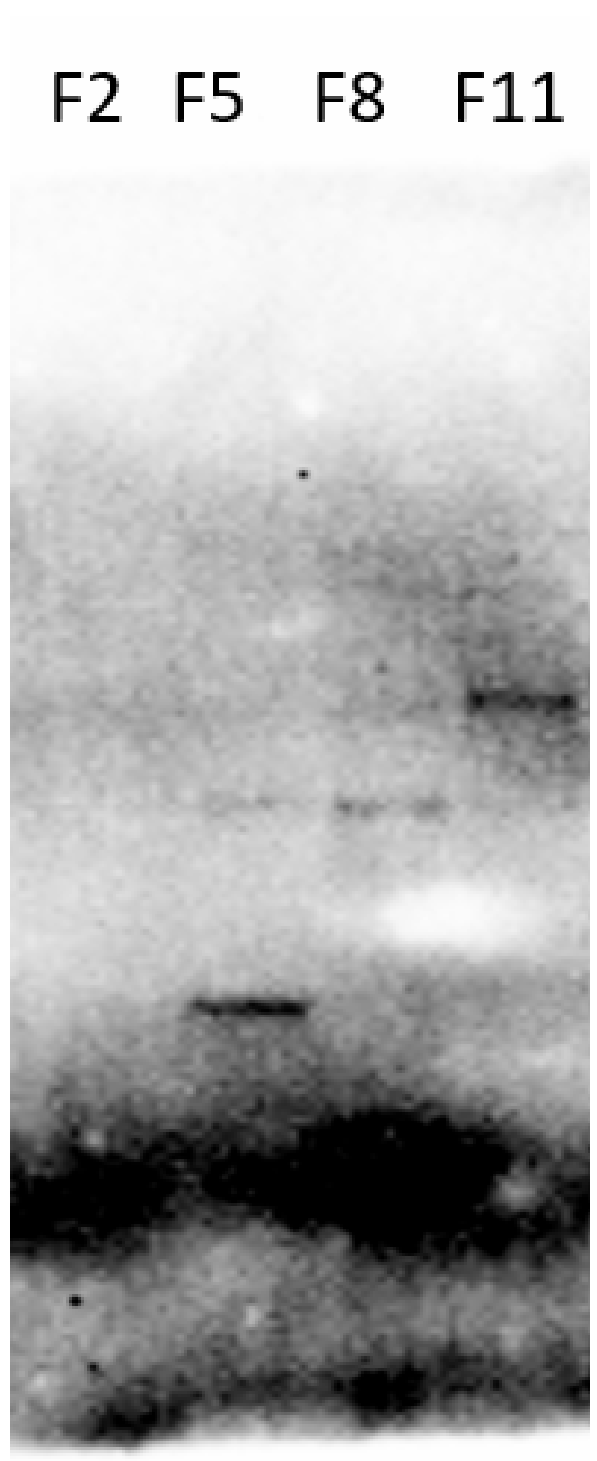

From Figure 3C, P8L/G172S

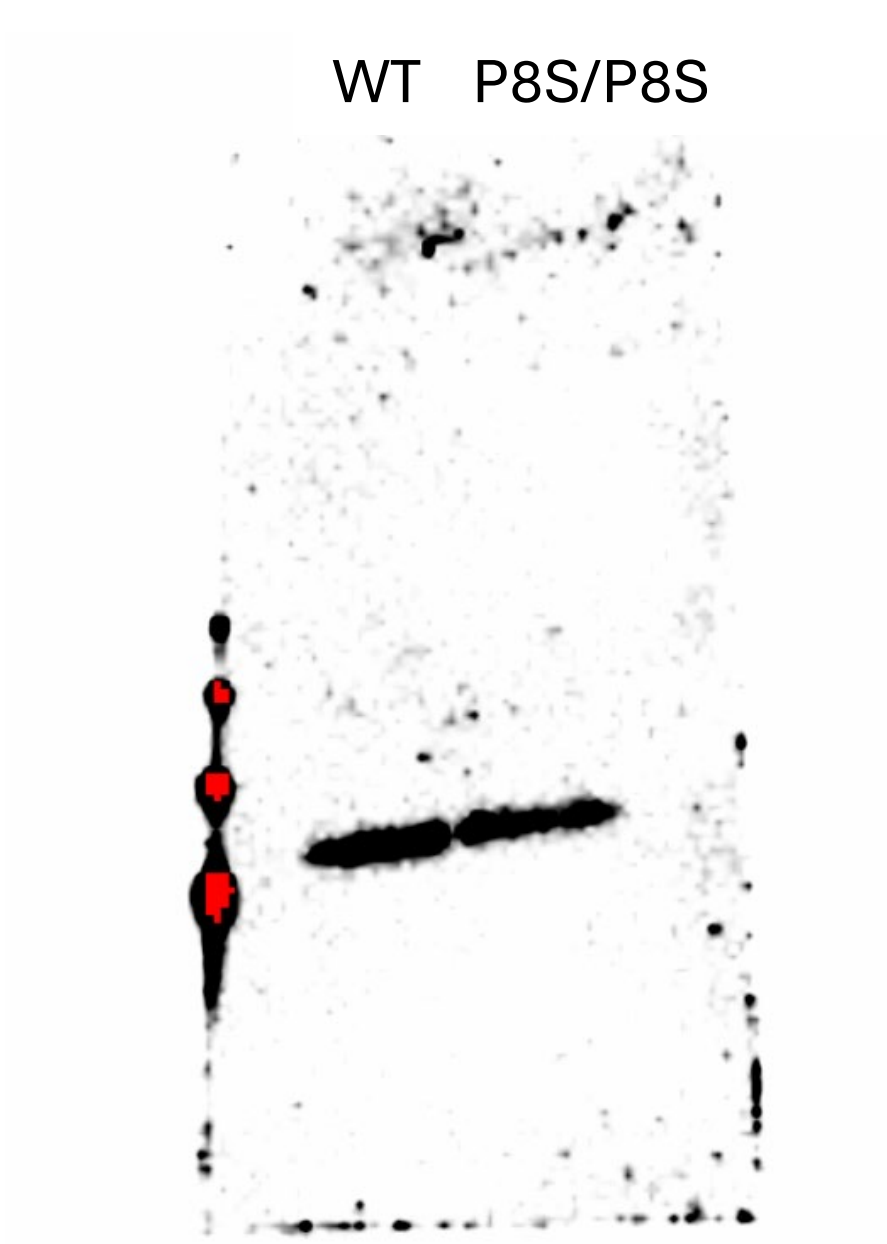

From Figure 6E

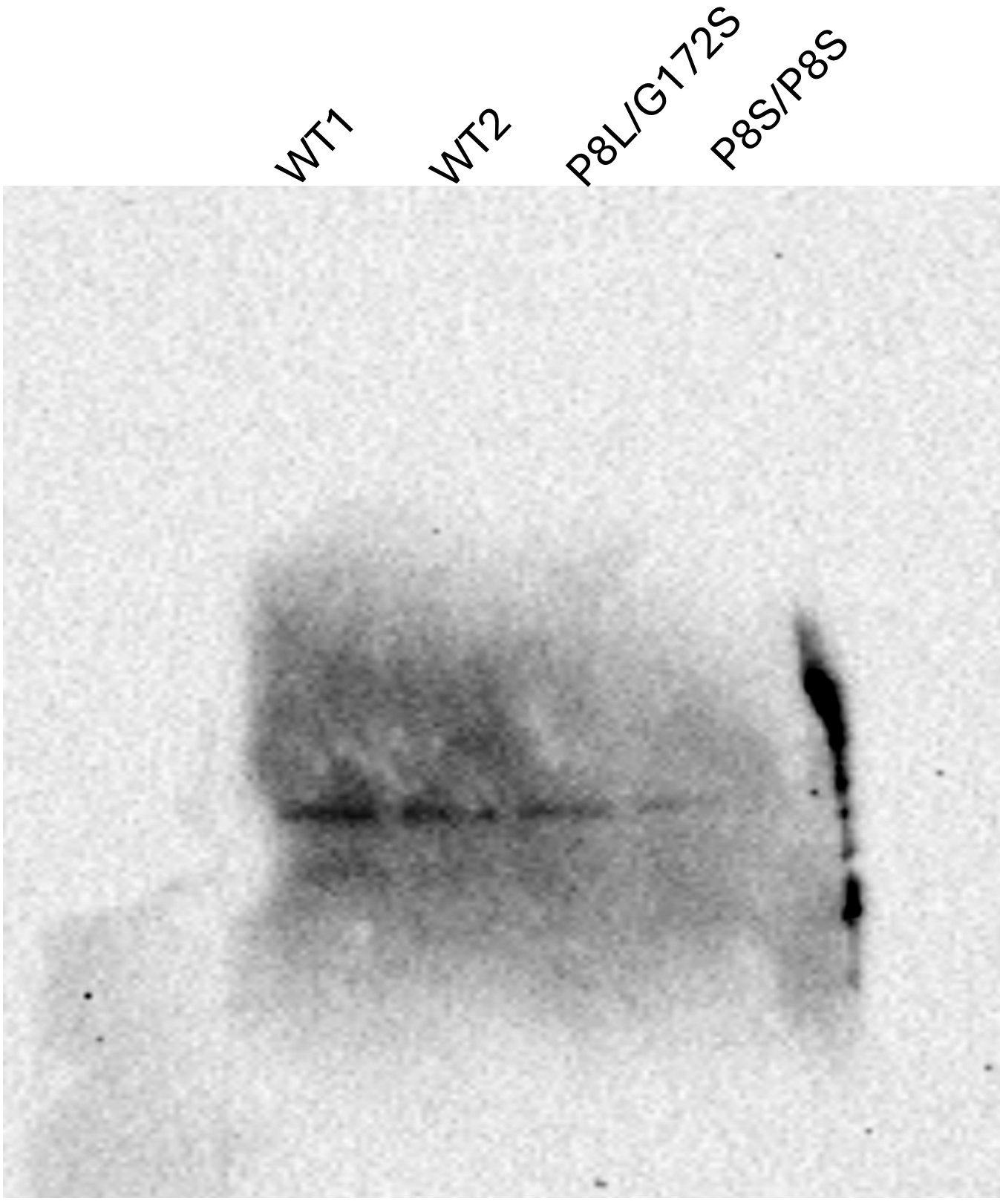

From Figure 7D, p62

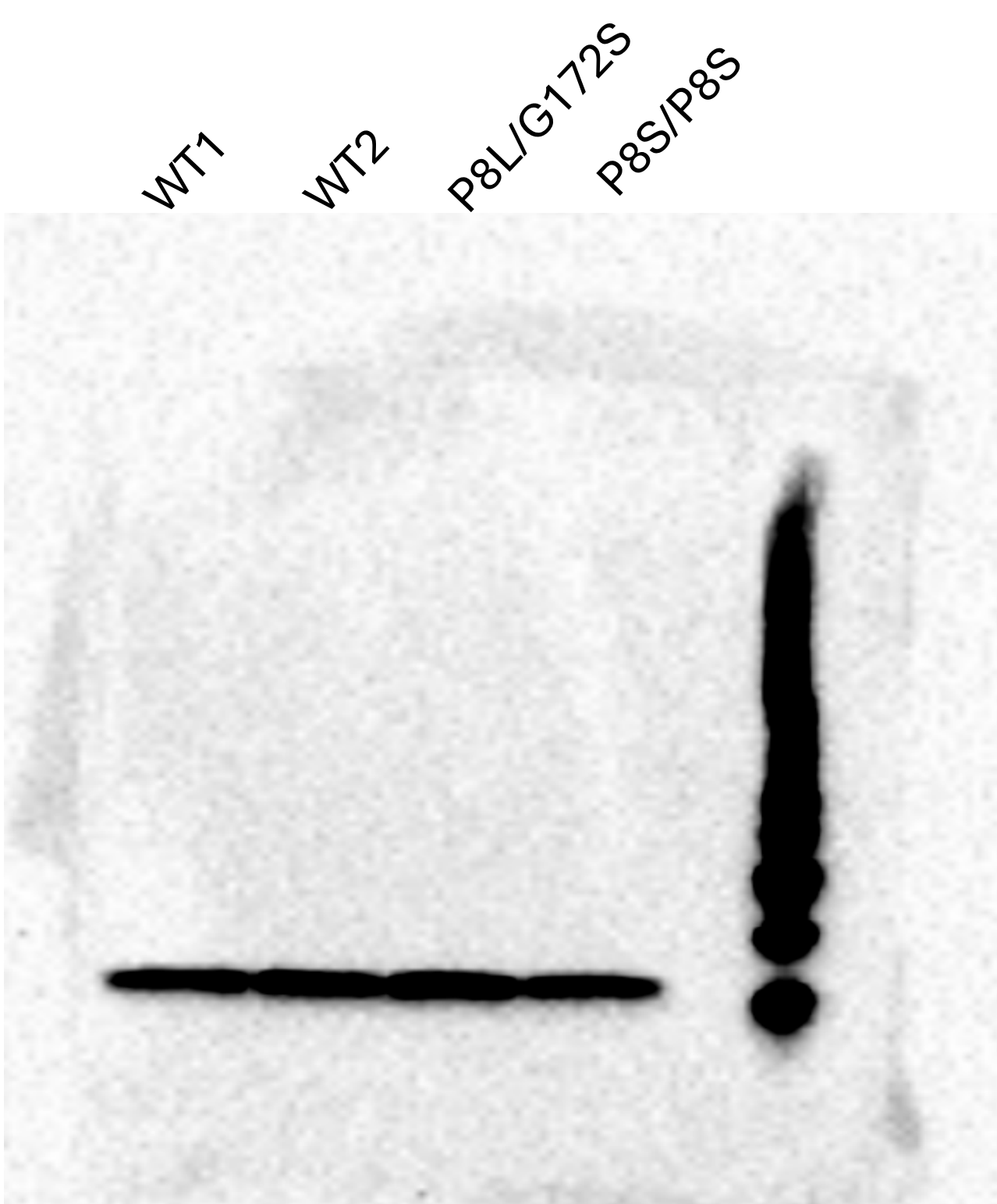

From Figure 7D, CD81
